# Secretomes of M1 and M2 macrophages decrease the release of neutrophil extracellular traps

**DOI:** 10.1038/s41598-023-42167-1

**Published:** 2023-09-20

**Authors:** Aneta Manda-Handzlik, Adrianna Cieloch, Weronika Kuźmicka, Agnieszka Mroczek, Anna Stelmaszczyk-Emmel, Urszula Demkow, Małgorzata Wachowska

**Affiliations:** 1 Department of Laboratory Diagnostics and Clinical Immunology of Developmental Age, Medical University of Warsaw, Zwirki i Wigury 63a Street, 02-091 Warsaw, Poland; 2 Doctoral School, Medical University of Warsaw, Zwirki i Wigury 61 Street, 02-091 Warsaw, Poland

**Keywords:** Cell biology, Immunology

## Abstract

The release of neutrophil extracellular traps (NETs) can be either beneficial or detrimental for the host, thus it is necessary to maintain a balance between formation and clearance of NETs. Multiple physiological factors eliciting NET release have been identified, yet the studies on natural signals limiting NET formation have been scarce. Accordingly, our aim was to analyze whether cytokines or immune cells can inhibit NET formation. To that end, human granulocytes were incubated with interleukin (IL)-4, IL-10, transforming growth factor beta-2 or adenosine and then stimulated to release NETs. Additionally, neutrophils were cultured in the presence of natural killer (NK) cells, regulatory T cells (Tregs), pro-inflammatory or anti-inflammatory macrophages (M1 or M2 macrophages), or in the presence of NK/Tregs/M1 macrophages or M2 macrophages-conditioned medium and subsequently stimulated to release NETs. Our studies showed that secretome of M1 and M2 macrophages, but not of NK cells and Tregs, diminishes NET formation. Co-culture experiments did not reveal any effect of immune cells on NET release. No effect of cytokines or adenosine on NET release was found. This study highlights the importance of paracrine signaling at the site of infection and is the first to show that macrophage secretome can regulate NET formation.

## Introduction

Neutrophils, effector cells of innate immune system, employ several strategies to fight infections. Among these mechanisms, the release of neutrophil extracellular traps (NETs) has been most recently discovered. NETs are composed of decondensed chromatin ornamented with microbicidal proteins, such as neutrophil elastase (NE), which are derived from neutrophil’s granules, nucleus or cytoplasm^1^. NETs serve as efficient traps to immobilize pathogens, prevent them from spreading and create a space with high local concentration of antimicrobial factors. Nevertheless, inadequate clearance or increased NET formation can contribute to the pathogenesis of multiple inflammatory conditions, e.g. cystic fibrosis or autoimmune diseases, such as systemic lupus erythematosus (SLE) and rheumatoid arthritis^2^. NETs involvement in these conditions is due to the presence of proteolytic proteins in their structure (e.g., NE, matrix metalopeptidase 9), which can damage adjacent tissues, as well as due to the exposure of intracellular self-antigens (e.g. double-stranded DNA, histones, peptide LL-37), which under normal conditions remain restricted from the recognition by immune cells^3,4^. Furthermore, NET formation primes cancer metastasis via immobilization of circulating tumor cells^5^. As a consequence, maintaining the balance between NET formation and degradation, as well as containment of excessive activation of neutrophils at the sites of inflammation, are of the utmost importance.

Multiple physiological inducers of NET release have been identified so far. It is well established that NETs are released in a response to bacterial, fungal, parasitic, and viral infections. Under in vitro conditions*,* neutrophils are triggered to release NETs by lipopolysaccharides (LPS), which are components of the outer membrane of Gram-negative bacteria; glucans and mannans derived from *Candida* species; as well as pro-inflammatory cytokines, such as interleukin (IL)-8 or tumor necrosis factor α^6^. When we consider the multiplicity of natural NETs inducers, threats associated with excessive, uncontrolled NET release, as well as the fact that most people do not develop autoimmune diseases in spite of multiple infections throughout the course of their lives, it is warranted to assume that neutrophils activation and NET formation in humans is tightly regulated. Yet, studies on natural signals limiting NET formation have been scarce. It was shown that phorbol 12-myristate 13-acetate (PMA)-induced NET release is inhibited by adenosine and IL-4, but otherwise, the influence of natural anti-inflammatory compounds on NET release remains largely unknown^7,8^.

Of note, the majority of in vitro studies on NET release in humans were conducted with the use of isolated neutrophils in mono-culture, whereas in vivo*,* neutrophils do not stand alone in the fight against microbes. Neutrophils are supported by a team of other immune cells, like dendritic cells, macrophages, lymphocytes, and natural killer (NK) cells, and this co-operation lays the groundwork for both the elimination of pathogens and the resolution of inflammation^9,10^. There is growing evidence that neutrophils can both sense the signals delivered by neighboring cells as well as shape adaptive and innate immune responses e.g. by secreting cytokines or via direct interactions with immune cells^11^. For example, it was shown that direct interaction of LPS-activated regulatory T cells (Tregs) with neutrophils can promote anti-inflammatory activity of the latter via induction of IL-10 synthesis^12^. Furthermore, macrophage secretome negatively influences neutrophil degranulation in mice^13^. Neutrophils’ functions can also be influenced by activated NK cells, which can affect survival rates, phagocytosis, synthesis of reactive oxygen species (ROS) and expression of surface markers^14^. However, only a few studies considered the impact of various immune cells on NET release. It was shown that murine NK cells induce NET synthesis via the secretion of IFN-γ^15^. Interestingly, studies on bovine *Mycobacterium* infection revealed that direct co-operation between neutrophils and macrophages allows for efficient bacteria killing and reduced inflammatory reaction, reflected in diminished NET formation and decreased IL-1β and IL-8 levels, as compared to the culture of either cell type alone^16^. It has been also shown that macrophages can affect NET release via the soluble mediators released into the bodily fluids. Yang et al. demonstrated that enhanced NET formation observed in SLE patients may be due to downregulated expression of micro RNA (miRNA) 4512 in monocytes and macrophages^17^. Besides aforementioned reports, we are not aware of any other studies deciphering the direct or indirect effect of various immune cells on NETting neutrophils.

In the light of incomplete knowledge on natural signals limiting NET formation, their identification became the aim of this project. We hypothesized that anti-inflammatory cytokines and other molecules, such as IL-10, IL-4, transforming growth factor beta (TGF-β), or adenosine^18^, may influence NET release. Furthermore, we speculated that activated immune cells may interact at the site of infection and inflammation, preventing excessive activation of neutrophils. Accordingly, we tested the hypothesis that NET release may be influenced by anti-inflammatory cytokines, adenosine, contact with activated immune cells (Tregs, NK cells or macrophages) or cultured media collected from these cells. In this study, we provide evidence that NET synthesis is not influenced by anti-inflammatory molecules, such as IL-4, IL-10, TGF-β or adenosine. However, we identify both anti- and pro-inflammatory macrophages as the source of soluble mediators significantly limiting NET release as well as the synthesis of reactive oxygen species (ROS) generated by activated neutrophils. In our studies, direct macrophage-neutrophil contact over the course of NET formation did not affect the process of NET release. Furthermore, neither Tregs nor NK cells alone nor their supernatants affected NET release. Overall, our study highlights the vital role of crosstalk between innate phagocyting cells, macrophages and neutrophils, as a factor regulating antimicrobial activity of neutrophils.

## Results

### NET release is not affected by IL-4, IL-10, TGF-β and adenosine

**Figure 1:**
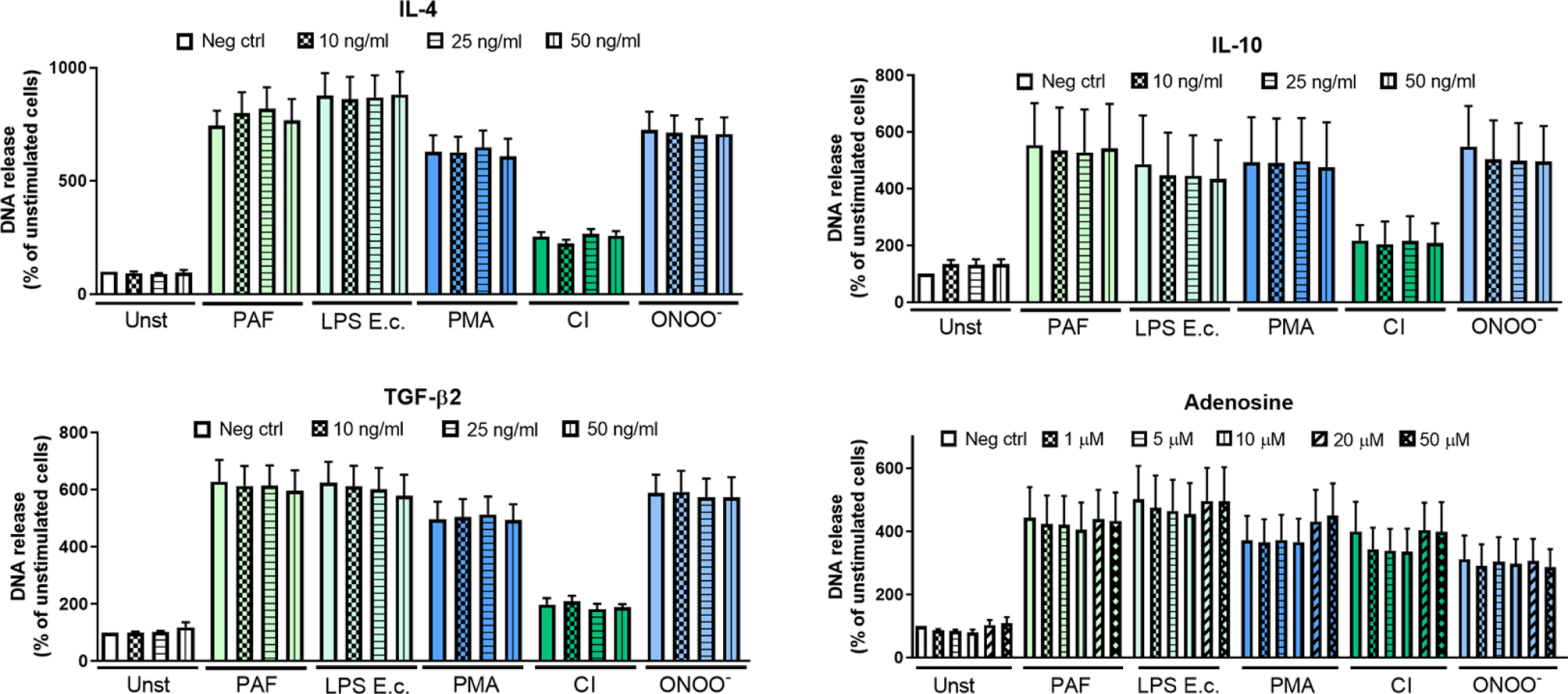
Interleukin 4, interleukin 10, transforming growth factor β nor adenosine do not affect NET release. Neutrophils were incubated with increasing concentrations of interleukin (IL)-4, IL-10, transforming growth factor β (TGF-β) or adenosine for 1 h. Neutrophils cultured without addition of cytokines constituted negative control (neg ctrl). The cells were then stimulated with 2.5 μM platelet–activating factor (PAF), 100 μM peroxynitrtite (ONOO^-^), 4 μM calcium ionophore A23187 (CI), 100 nM phorbol 12-myristate 13-acetate (PMA) or 5 μg/ml lipopolysaccharides (LPS) isolated from *E. coli*. After 3 h, the amount of DNA released was measured fluorometrically. N = 6, *n* the number of biological replicates, the data were analyzed with 1-way ANOVA with *post-hoc* Dunn’s or Holm-Sidak’s multiple comparisons test (depending on the normality of data), no statistically significant differences of NET release in the presence of cytokines/adenosine were found.

The course and intensity of immune response is regulated by the interplay between pro- and anti-inflammatory factors. The functions of immune effector cells can be suppressed by multiple cytokines, with major contributors such as IL-4, IL-10 and TGF-β^19^, as well as by other classes of molecules, including purine nucleosides, with adenosine as an example^20,21^. Accordingly, we hypothesized that NET release may be negatively influenced by aforementioned anti-inflammatory agents. To test this hypothesis, we incubated human neutrophils with increasing concentrations of IL-4, IL-10, TGF-β, and adenosine, and then stimulated the cells with a wide range of inducers, employing various molecular mechanisms^22,23^ to trigger NET release: platelet-activating factor (PAF), LPS isolated from *E. coli*, PMA, calcium ionophore A23187 (CI) or peroxynitrite (ONOO^−^). The amount of DNA released by NETting cells was measured fluorometrically and the process of NET release was visualized following immunostaining. As expected, each of the stimuli induced NET release, with CI being least efficient under tested conditions (Fig. 1). Nevertheless, NET formation remained unaltered in the presence of adenosine or any of the tested cytokines (Fig. 1, Supplementary Fig. 1).

### Secretome of M1 and M2 macrophages negatively regulates NET formation and synthesis of reactive oxygen species by neutrophils

Macrophages are tissue-resident, primary innate cells of diverse functions, which are reflected in the wide spectrum of macrophage phenotypes. Macrophages are traditionally divided into M1 (pro-inflammatory) and M2 (anti-inflammatory) phenotypes, with M2 further divided into several subtypes (M2a, M2b, M2c, M2d), depending on the secreted cytokines, stimuli and transcriptional profile^24^. Since neutrophils and macrophages have been shown to cooperate during an immune response^25^, we investigated whether NET release may be regulated by direct contact of neutrophils with macrophages or secretome released by polarized macrophages. To that end, we isolated monocytes from peripheral blood and differentiated them into macrophages of proinflammatory or anti-inflammatory phenotype. To acquire M1 macrophage phenotype, monocytes were differentiated with granulocyte–macrophage colony-stimulating factor (GM-CSF) and further polarized with interferon (IFN)-γ. To acquire M2 macrophage phenotype, monocytes were differentiated with macrophage colony-stimulating factor (M-CSF) and further polarized with IL-4 (M2a macrophages) or IL-10 (M2c macrophages). The overview of the differentiation protocol and the procedure of supernatant collection is shown in Supplementary Fig. 2a. Polarization of monocytes into M1 macrophages was confirmed by the following expression of antigens: CD68^+^/CD80^+^/CD163^−/low^, whilst M2 macrophages were characterized by CD68^+^/CD80^−/low^/CD163^+^ immunophenotype (Supplementary Fig. 2b). Polarized cells were subsequently co-cultured with neutrophils. Alternatively, supernatants from macrophage cultures were collected and added to neutrophils prior to cells’ stimulation.

**Figure 2:**
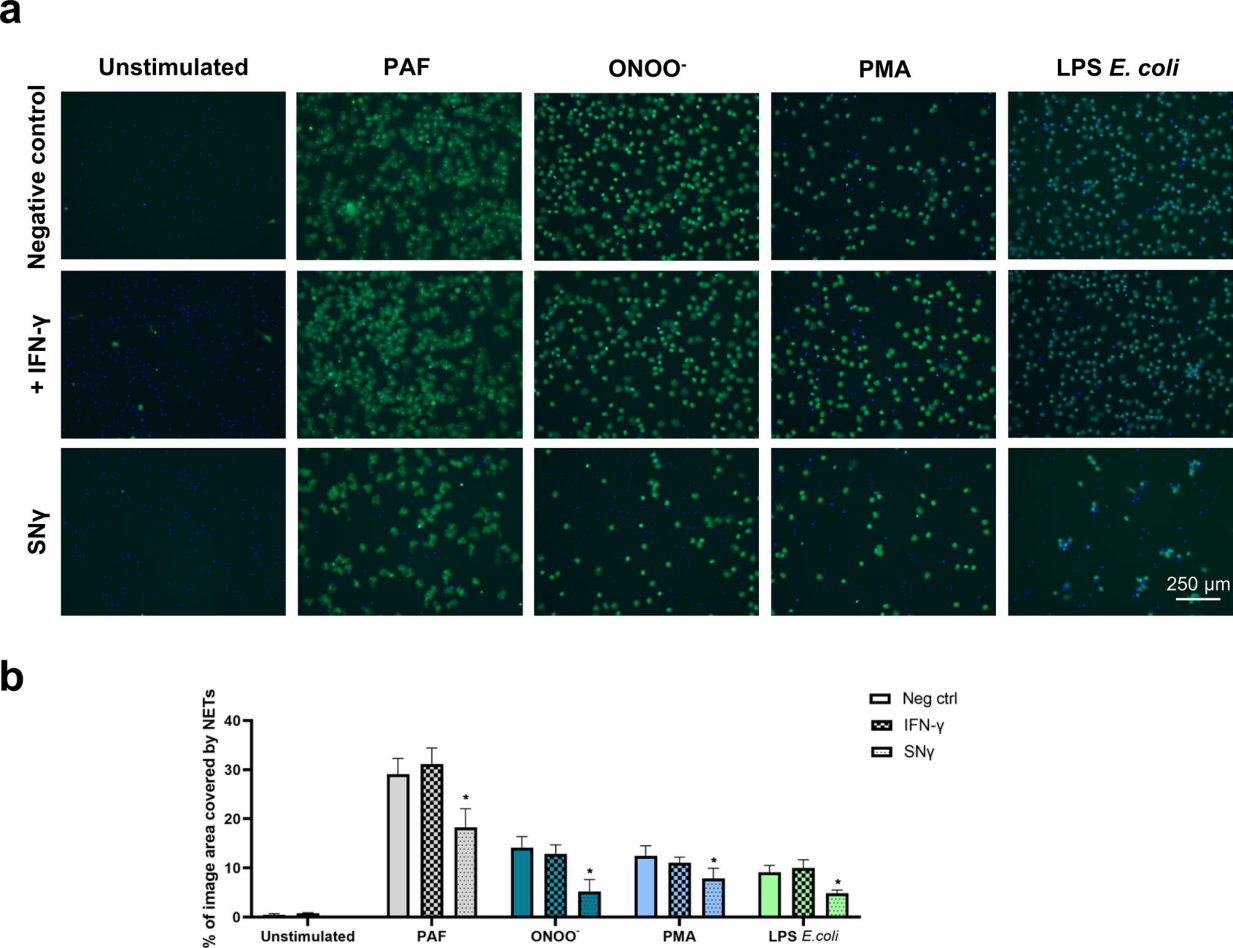
Secretome of M1 macrophages decreases NET release. Neutrophils were incubated for 1 h with supernatant collected from monocytes polarized into M1 macrophages. This supernatant contained 50 ng/ml IFN-γ that was used for the polarization of macrophages, and was abbreviated as SNγ. As controls, neutrophils were incubated in medium alone (negative control, neg ctrl) or in medium with IFN-γ. Subsequently, neutrophils were stimulated with 2.5 μM PAF, 100 μM ONOO^−^, 100 nM PMA or 5 μg/ml LPS isolated from *E. coli*. NET formation was assessed after 3 h. To visualize NETs, DNA was stained with 1.25 µg/ml Hoechst 33342 (blue) and 100 nM SYTOX Green (green) and microscopical images were taken. Percentage coverage of image area by NETs was assessed with the use of PartSeg software v0.13.14 with Trapalyzer Plugin. Routinely, two or three images at the magnification of 10 × were taken per each condition for each biological replicate. N = 8. (**a**) Representative images are shown. (**b**) Means + SEM are shown, for each stimulus the data were analyzed by one-way ANOVA with *post-hoc* Holm-Sidak’s multiple comparisons test (LPS *E. coli*) *vs* neg ctrl or by Friedman test with *post-hoc* Dunn’s multiple comparisons test (unstimulated (Unst), PAF, ONOO^-^, PMA**)** *vs* neg ctrl. *P ≤ 0.05.

First, we analyzed the influence of the secretome of proinflammatory macrophages on NET release. Neutrophils were cultured in the presence of the supernatant collected from M1 macrophages, containing IFN-γ. As a control, neutrophils were incubated in medium alone or in medium with IFN-γ. As NETs inducers, we used PAF, ONOO^−^, PMA, and LPS isolated from *E. coli*. Microscopic live imaging analyses revealed that in the presence of macrophage supernatant, the amount of cloud-like DNA structures was lower than in samples incubated with culture medium alone and the same effect was not observed in the presence of IFN-γ, which proved that the inhibition of NET release was not dependent on the presence of IFN-γ in macrophage supernatant (Fig. 2a,b). Although NET release was inhibited in the presence of M1 macrophage supernatant, the inhibition was not complete and NET release in the presence of supernatant did not reach as low levels as in unstimulated cells.

**Figure 3:**
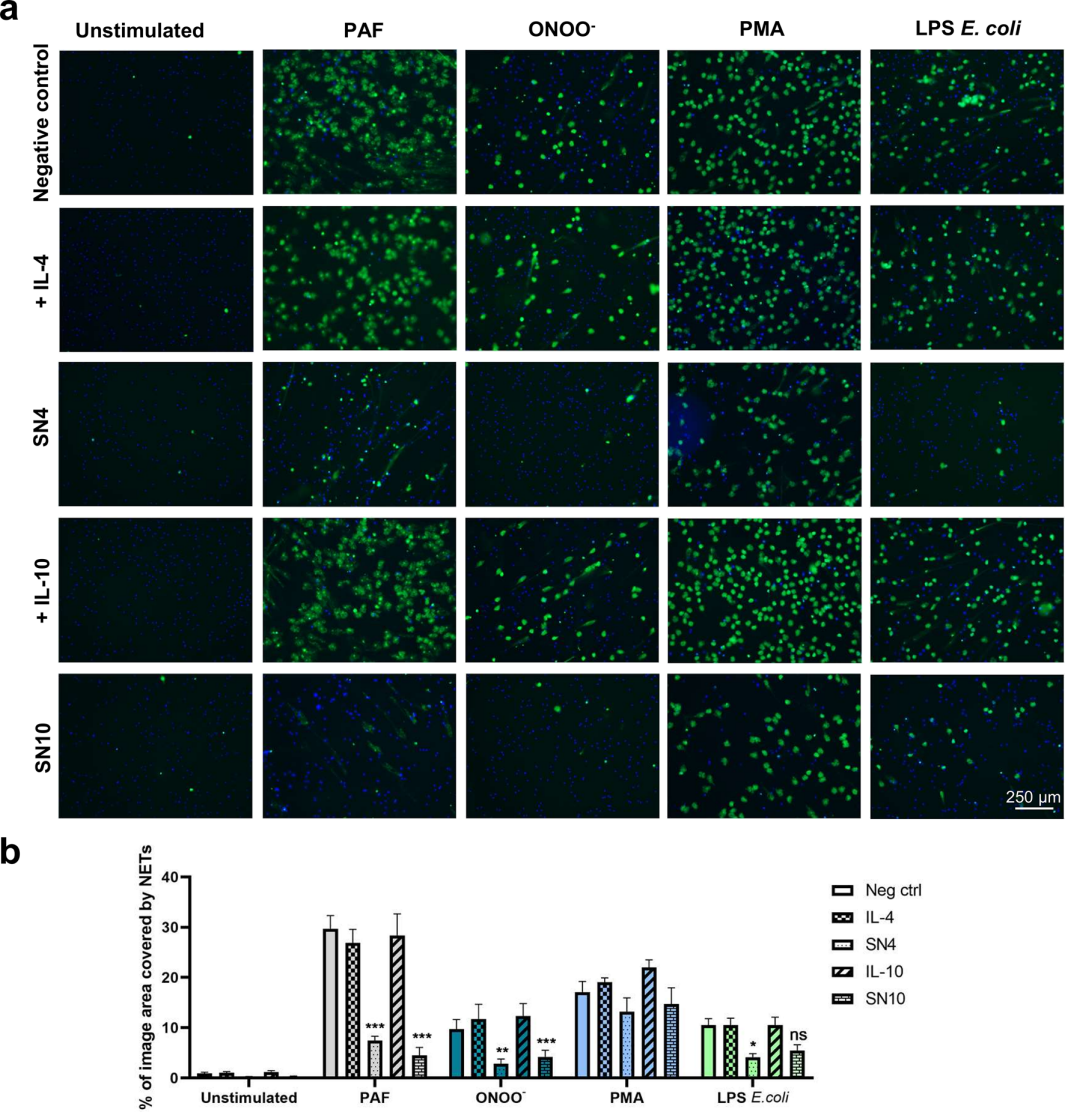
Secretome of M2 macrophages decreases NET release. Neutrophils were incubated for 1 h with supernatant collected from monocytes differentiated into M2a or M2c macrophages. Collected supernatants contained cytokines that were used for the polarization of macrophages, i.e. 20 ng/ml IL-4 (M2a) or IL-10 (M2c) and were abbreviated respectively as SN4 or SN10. As controls, neutrophils were incubated in medium (negative control, neg ctrl) or in medium with IL-4 or IL-10. Subsequently, neutrophils were stimulated with 2.5 µM PAF, 100 µM ONOO^−^, 100 nM PMA or 5 μg/ml LPS isolated from *E. coli*, NET formation was assessed after 3 h. To visualize NETs, DNA was stained with 1.25 µg/ml Hoechst 33342 (blue) and 100 nM SYTOX Green (green) and microscopical images were taken. Percentage coverage of image area by NETs was assessed with the use of PartSeg software with Trapalyzer Plugin. Routinely, two or three images at the magnification of 10 × were taken per each condition for each biological replicate; n = 5 (PMA) or n = 7 (unstimulated, PAF, ONOO^−^, LPS *E. coli*). (**a**) Representative images are shown. (**b**) Means + SEM are shown, for each stimulus the data were analyzed *vs* negative control by one-way ANOVA with *post-hoc* Holm-Sidak’s multiple comparisons test (PAF, ONOO^−^, LPS *E. coli*) or by Friedman test with *post-hoc* Dunn’s test when appropriate (unstimulated, PMA). ^Ns^P > 0.05, *P ≤ 0.05, **P ≤ 0.01, ***P ≤ 0.001.

Similarly, supernatant collected from anti-inflammatory, M2a or M2c, macrophages significantly inhibited NET formation upon stimulation with PAF and ONOO^−^ (Fig. 3a,b). Furthermore, LPS-induced NET release was diminished in the presence of supernatant collected from macrophages polarized with the use of IL-4 (M2a macrophages). Similar, inhibitory influence on LPS-induced NET release was observed for the supernatant collected from macrophages polarized with the use of IL-10 (M2c macrophages), nevertheless this effect was not statistically significant (p = 0.2076). PMA-induced NET release was slightly diminished in the presence of M2a or M2c macrophage supernatant but this trend did not reach statistical significance (p = 0.1364). The lack of inhibitory effect of IL-4 or IL-10 on NET release confirmed that constituents other than IL-4 or IL-10 are responsible for the inhibitory effect of M2 macrophages secretome on NET release.

**Figure 4:**
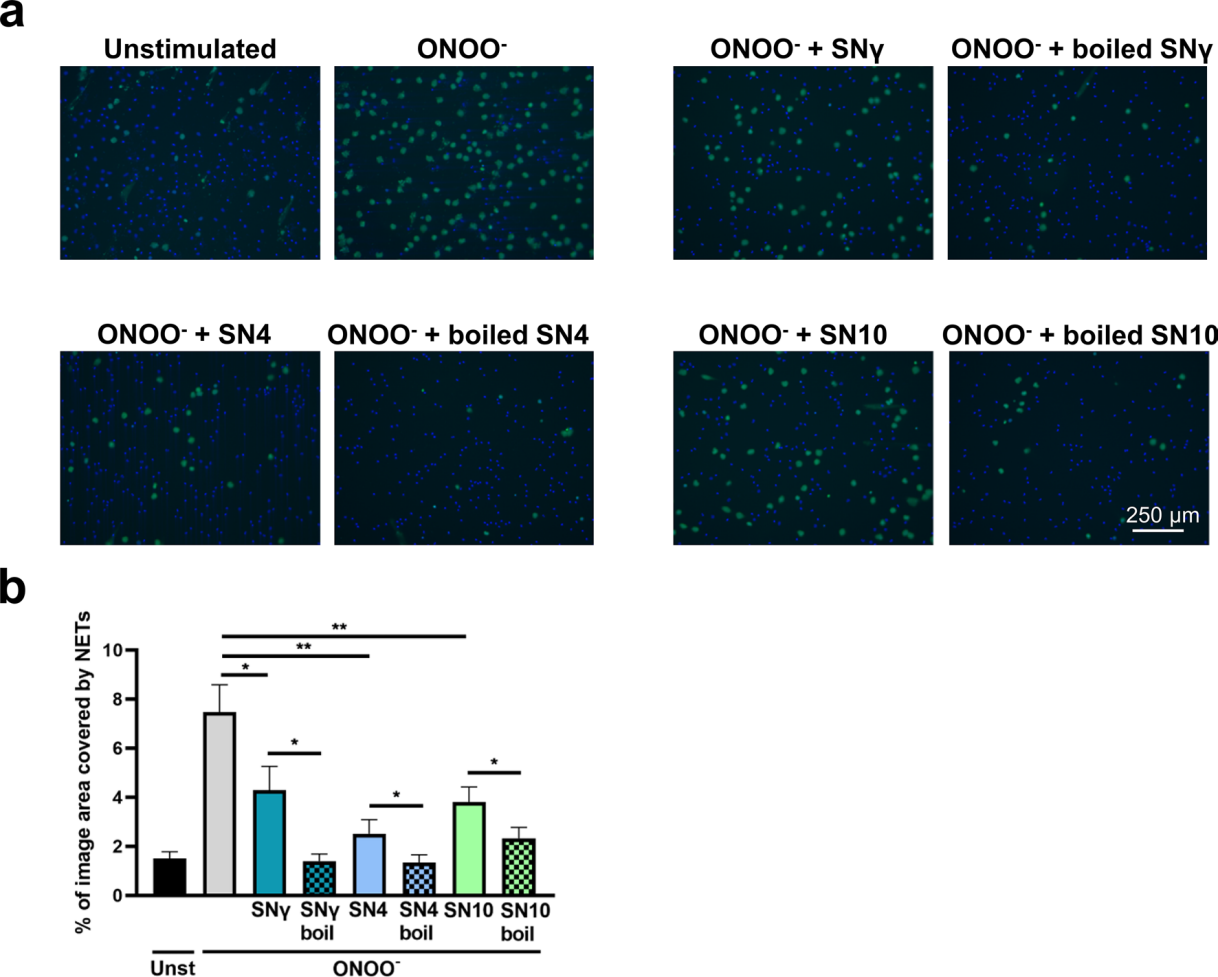
Boiling of macrophage secretome does not prevent the inhibition of NET formation. Neutrophils were incubated for 1 h with supernatant collected from monocytes differentiated into M1, M2a or M2c macrophages. Collected supernatants contained cytokines that were used for the polarization of macrophages, i.e. 50 ng/ml IFN-γ (M1), 20 ng/ml IL-4 (M2a) or IL-10 (M2c) and were abbreviated as SNγ, SN4 or SN10, respectively. When indicated, supernatant was boiled (95 °C, 10 min) prior to the addition to the cells. Alternatively, neutrophils were incubated in medium. Subsequently, neutrophils were stimulated with 100 µM ONOO^-^ and NET formation was assessed after 3 h. To visualize NETs, DNA was stained with 1.25 µg/ml Hoechst 33,342 (blue) and 100 nM SYTOX Green (green) and microscopical images were taken. Percentage coverage of image area by NETs was assessed with the use of PartSeg software with Trapalyzer Plugin. Routinely, at least three images at the magnification of 10 × were taken per each condition for each biological replicate; n = 8. (**a**) Representative images are shown. (**b**) Means + SEM are shown, the data were analyzed by one-way ANOVA with *post-hoc* Holm-Sidak’s multiple comparisons test. *P ≤ 0.05, **P ≤ 0.01.

Subsequently, we investigated whether boiling of the supernatants collected from M1, M2a and M2c macrophages diminishes their NET-inhibiting properties. It served as a rudimentary means of analyzing, whether factor(s) diminishing NET release are proteins. Surprisingly, boiling of the supernatant not only failed to revert NET-inhibiting properties of macrophage supernatants, but even caused further inhibition of NET formation (Fig. 4). It suggests that molecule(s) found in macrophage secretome, responsible for the inhibition of NET formation, are heat stable, which precludes the involvement of proteins. On the other hand, denaturation of heat-labile factors present in collected supernatant allowed for even more efficient reduction of NET formation in the presence of macrophage supernatant. This may suggest that macrophage secretomes represent a mixture of various factors of opposing influence on NET release. Alternatively, denatured proteins may form aggregates which inhibit NET release.

Notably, NET-decreasing properties of macrophage supernatant were limited to polarized macrophages. Supernatant collected from unpolarized macrophages (derived from monocytes cultured for 7 days in full medium, without addition of growth hormones or cytokines) did not exert similar inhibitory effect on NET release as M1, M2a or M2c secretome (Supplementary Fig. 3a,b). Among supernatants collected from polarized macrophages, secretome of M2a macrophages more potently diminished NET formation than secretomes of M1 and M2c macrophages. When testing various dilutions of supernatants, we found that only supernatant collected from macrophages M2a (SN4) retained its ability to inhibit ONOO^-^-induced NET release following 1:2 dilution with medium (Supplementary Fig. 4a,b). Further dilutions (1:5 and 1:10) of supernatants collected from M1, M2a and M2c macrophages did not diminish NET formation.

**Figure 5:**
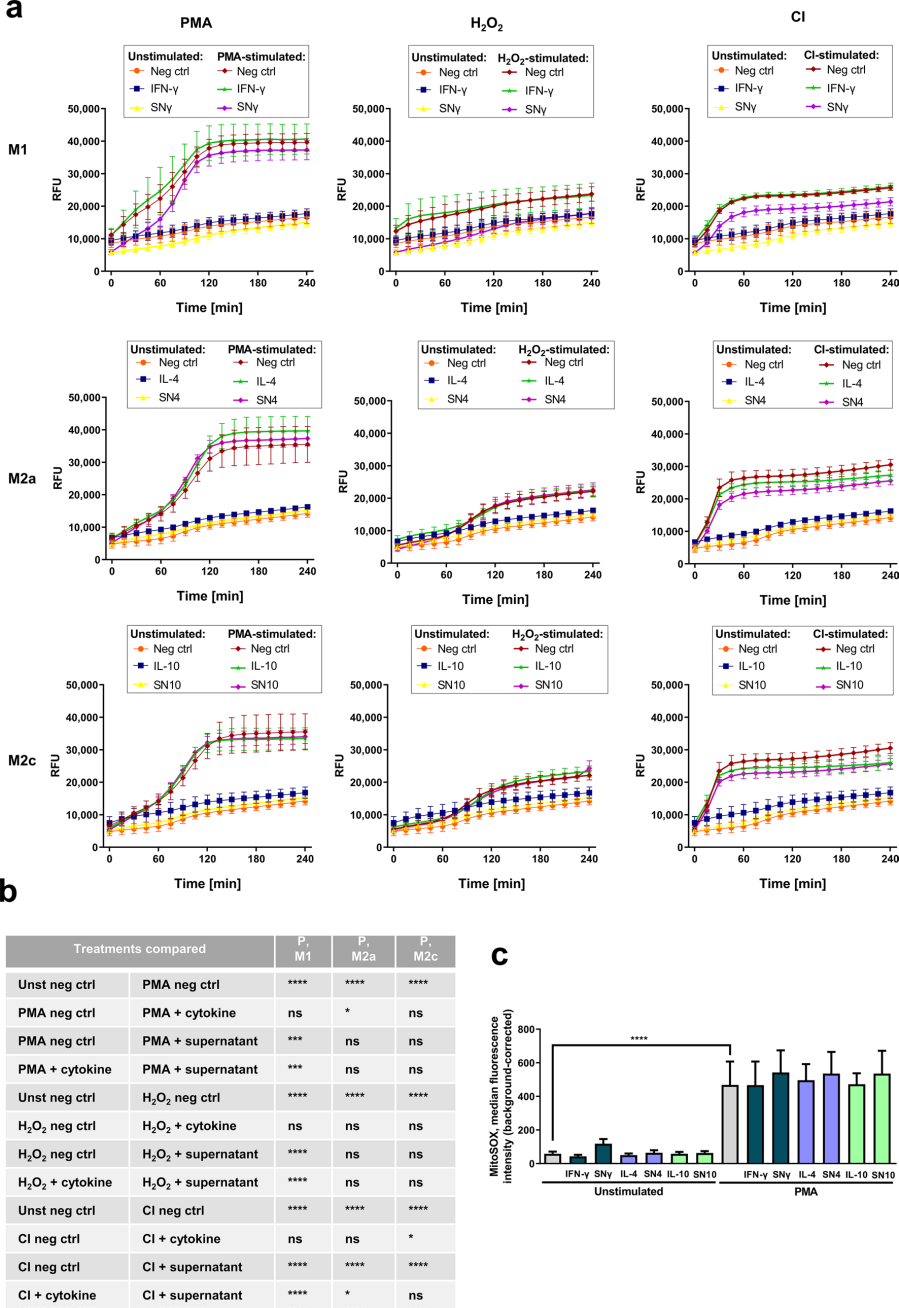
Secretome of M1 and M2 macrophages diminishes ROS synthesis by neutrophils. Neutrophils were incubated for 1 h with supernatant collected from monocytes differentiated into M1, M2a or M2c macrophages. Collected supernatants contained cytokines that were used for the polarization of macrophages, i.e. 50 ng/ml IFN-γ (M1), 20 ng/ml IL-4 (M2a) or IL-10 (M2c) and were abbreviated as SNγ, SN4 or SN10, respectively. As controls, neutrophils were incubated in medium (negative control, neg ctrl) or in medium with IFN-γ, IL-4 or IL-10. (**a,b**) To induce ROS synthesis, neutrophils were stimulated with 4 µM CI, 100 nM PMA or 100 µM H_2_O_2_. ROS synthesis was monitored fluorometrically for 4 h in neutrophils loaded with DHR. (**c**) Synthesis of mitochondrial ROS was analyzed with a flow cytometer after 60-min stimulation with PMA and staining of the cells with MitoSOX. (**a**) Means ∓ SEM are shown, n = 8 for M1, n = 6 for M2a and M2c; (**b**) the data presented in (**a**) were analyzed with 2-way ANOVA with Tukey’s multiple comparisons *post-hoc* test, the table presents P values for each type of polarized macrophages. (**c**) For background correction, median fluorescence intensity (MFI) of the cells which were not loaded with MitoSOX was subtracted from the MFI of the cells loaded with MitoSOX; the image presents means + SEM, n = 5. The data were analyzed with 2-way ANOVA with *post-hoc* Šídák’s multiple comparisons test. ^Ns^P > 0.05, *P ≤ 0.05, ***P ≤ 0.001, ****P ≤ 0.0001. *RFU* relative units of fluorescence.

It was previously shown that one of the key NET-related mechanisms is the synthesis of reactive oxygen species (ROS). ROS necessary for NET release are either formed in a reaction catalyzed by NADPH oxidase or they are derived from other sources, e.g. mitochondria^26^. Based on these premises, we further investigated whether NETs-suppressing activity of macrophage supernatant may be due to its ROS-inhibiting properties. First, we cultured neutrophils in the presence of macrophage supernatants and analyzed ROS synthesis with the use of dihydrorhodamine (DHR) 123, a fluorescent probe sensitive to hydrogen peroxide (H_2_O_2_) and peroxynitrite. As inducers of oxidative burst we used PMA, CI and H_2_O_2_. We found that M1 macrophage secretome significantly inhibited oxidative burst induced by either of the stimuli, and this effect was not observed for IFN-γ alone (Fig. 5a,b). These observations imply that constituents other than IFN-γ, found in M1 macrophage secretome, diminish ROS synthesis by neutrophils. We also performed similar experiments regarding the influence of macrophage secretome on ROS synthesis for anti-inflammatory, M2 macrophages. Interestingly, the inhibitory effect of macrophage secretome on oxidative burst of neutrophils was observed for M2a macrophages only when CI was used as an inducer (Fig. 5a,b). IL-10 alone inhibited the oxidative burst of neutrophils and in the presence of M2c macrophages supernatant, containing IL-10, this effect was not further enhanced.

To analyze whether macrophage supernatant can negatively influence the synthesis of mitochondrial ROS, we stained the neutrophils with MitoSOX, a fluorescent mitochondrial superoxide indicator. The synthesis of mitochondrial superoxide by neutrophils was enhanced upon stimulation with PMA, but we did not observe any alterations in mitochondrial ROS synthesis in the presence of supernatants collected from M1, M2a or M2c macrophages (Fig. 5c).

**Figure 6:**
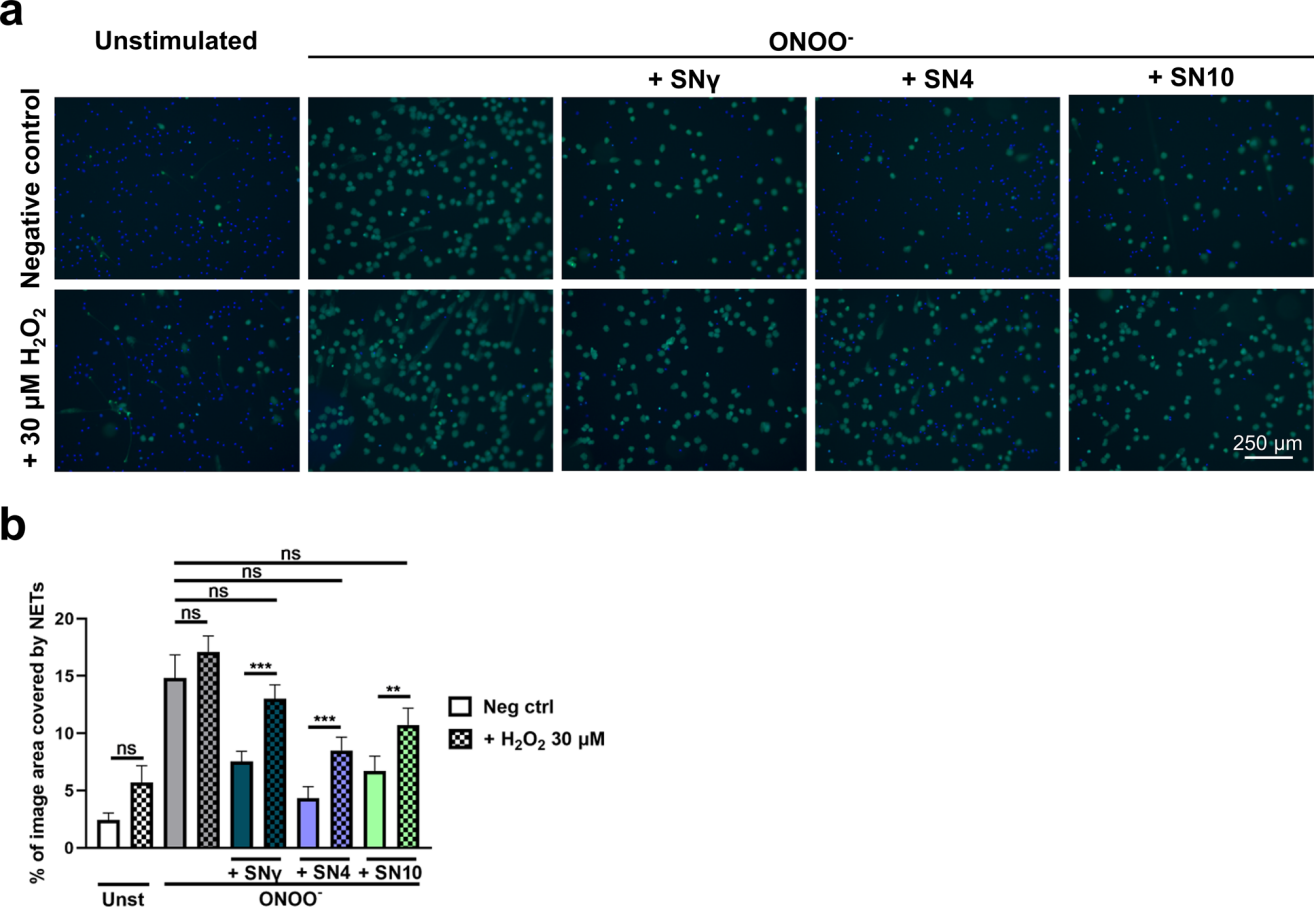
External source of ROS reverts inhibitory effect of macrophage supernatant on NET release. Neutrophils were incubated for 1 h with supernatant collected from monocytes differentiated into M1, M2a or M2c macrophages (i.e. SNγ, SN4 or SN10, respectively). Subsequently, neutrophils were stimulated to release NETs with 100 µM ONOO^−^. When indicated, 30 µM H_2_O_2_ was added as an external source of ROS; medium constituted negative control (neg ctrl). NET release was assessed after 3 h. To visualize NETs, DNA was stained with 1.25 µg/ml Hoechst 33342 (blue) and 100 nM SYTOX Green (green) and microscopical images were taken. Percentage coverage of image area by NETs was assessed with the use of PartSeg software with Trapalyzer Plugin. Routinely, at least three images at the magnification of 10 × were taken per each condition for each biological replicate, n = 8. (**a**) Representative images are shown. (**b**) Means + SEM are shown, the data were analyzed by one-way ANOVA with *post-hoc* Šídák’s multiple comparisons test. *Unst* unstimulated cells. ^Ns^P > 0.05, **P ≤ 0.01, ***P ≤ 0.001.

Next, we investigated whether the macrophage secretome-mediated reduction of NET formation would be reverted in the presence of external source of ROS. To that end, we incubated neutrophils with secretome collected from M1, M2a and M2c macrophages and then stimulated NET release with ONOO^-^ in the presence or absence of H_2_O_2_. H_2_O_2_ in the concentration used in this study (30 μM) only slightly increased NET formation by neutrophils not stimulated with ONOO^-^ and this increase was not statistically significant (Fig. 6a,b). However, addition of H_2_O_2_ as an external source of ROS to samples pretreated with M1, M2a or M2c secretome and then stimulated with ONOO^−^, significantly increased NET formation, abrogating the inhibitory effect of macrophage secretome (Fig. 6a,b).

**Figure 7:**
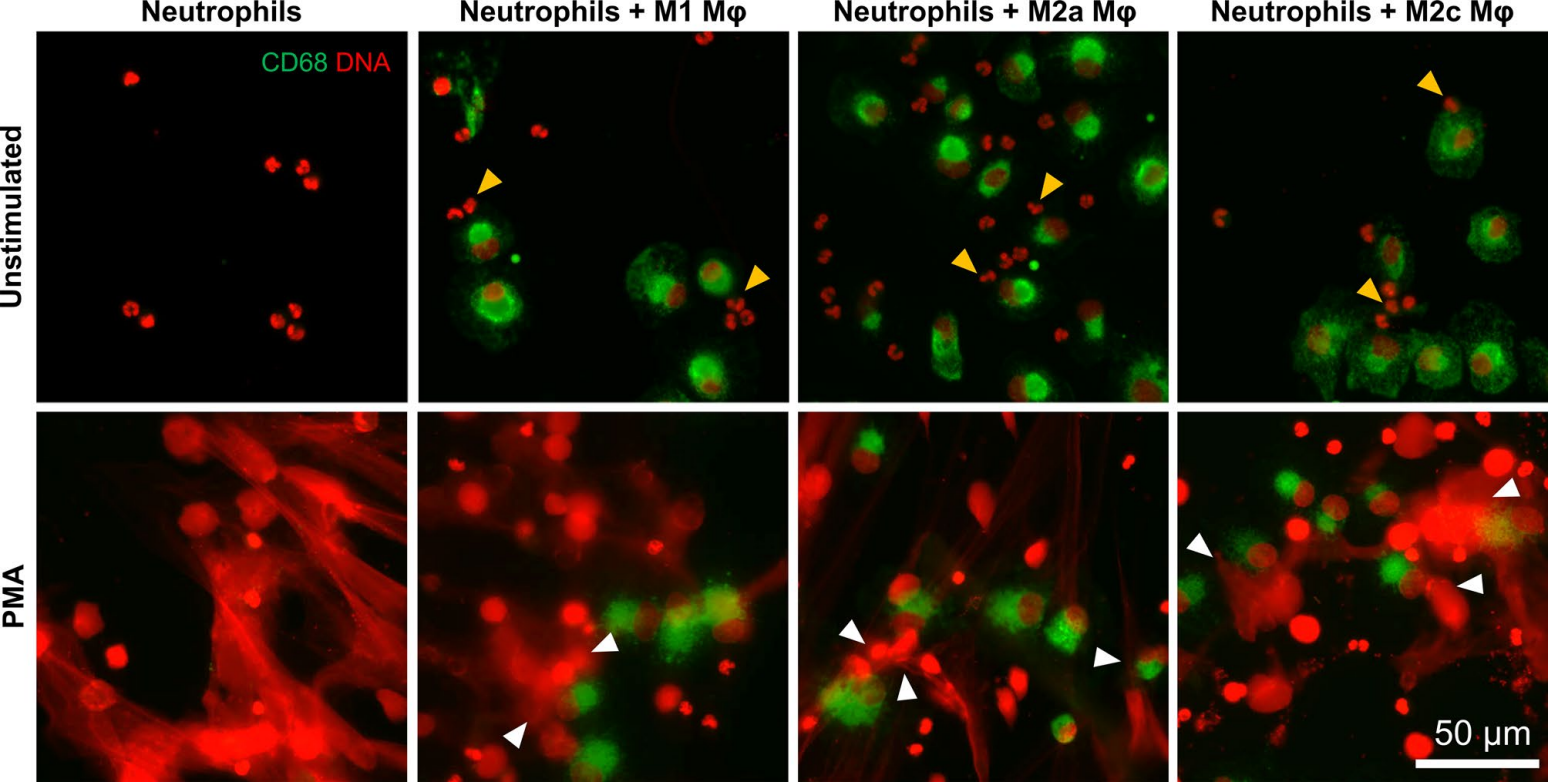
PMA-induced NET release is not inhibited by direct contact of neutrophils with macrophages. Neutrophils were co-cultured with monocyte-derived macrophages (at the ratio of 2:1 neutrophils:macrophages) for 1 h and subsequently neutrophils were stimulated with 100 nM PMA to release NETs. Three hours post stimulation the samples were fixed with paraformaldehyde and macrophages were immunostained with anti-CD68 antibody (green color) and DNA was counterstained with SYTOX Orange (red). Yellow arrowheads point to multilobulated granulocytes adjacent to macrophages. White arrowheads point to NET-derived DNA adjacent to macrophages. Images are representative for five biological replicates. *Mφ* macrophages.

Finally, we analyzed whether a direct contact of neutrophils and macrophages may affect NET release. In these studies, neutrophils were added to the wells of the plates with previously-polarized macrophages and solely PMA was used as an inducer. Other inducers were not applied, since remnants of FBS from the cell culture of macrophages inhibited their activity, as described previously^27^. In our studies, we did not observe the inhibition of PMA-induced NET release by neutrophils adjacent to macrophages (Fig. 7, white arrowheads point to NET-derived DNA adjacent to macrophages). Similarly, microscopical inspection of the samples did not reveal the overall NET release inhibition in the wells containing macrophages. Notably, we also did not observe an induction of NET release by neutrophils directly contacting polarized M1, M2a or M2c macrophages (Fig. 7, yellow arrowheads point to multilobulated granulocytes adjacent to macrophages).

### Regulatory T cells do not influence NET release, neither directly nor via the secreted factors

Regulatory T cells (Tregs) are a major subpopulation of immune cells of potent suppressive properties, which can affect the functions of multiple types of innate immunity cells, including neutrophils^12^. In our studies, we investigated whether activated Tregs may be the source of an immunosuppressive signal for NETs-releasing granulocytes. To study this, we isolated Tregs from peripheral blood of healthy volunteers and activated them with ImmunoCult™ Human CD3/CD28/CD2 T Cell Activator, a reagent containing soluble antibody complexes that bind to and cross-link CD3, CD28, and CD2 cell surface ligands, providing the required primary and co-stimulatory signals for T cell activation. Medium for cell culture of Tregs was supplemented with IL-2. The overview of the activation protocol and the procedure of supernatant collection is shown in Supplementary Fig. 5a. Activation and expansion of Tregs was confirmed microscopically as the presence of aggregates of activated cells (Supplementary Fig. 5b). Additionally, we analyzed the presence of very early activation marker, CD69, which is transiently expressed on activated T cells^28^. Under our experimental conditions, an increase in CD69 expression was observed only in Tregs isolated from 1 out of 3 healthy volunteers recruited into the study, which might result from the time-point chosen for cytometric analyses (Supplementary Fig. 5c).

Conversely to what we observed when studying the influence of macrophages supernatants on NET release, supernatant collected from expanded Tregs had no effect on NET release induced with PAF, PMA, ONOO^−^ or LPS *E. coli* (Supplementary Fig. 6a,b). IL-2, which was added to Tregs cell cultures and was present in supernatants collected from Tregs, had no effect on NET formation. Furthermore, we did not observe any differences in the level of NET formation between samples containing neutrophils alone or neutrophils co-cultured with Tregs (Supplementary Fig. 7). These analyses suggested that immunosuppressive properties of Tregs are not reflected in their influence on NET release.

### Natural killer cells do not influence NET release, neither directly nor via the secreted factors

NK cells, a subtype of innate lymphoid cells, represent a critical arm of immune response in cancer immunosurveillance and containment of viral infections. Notably, there is strong evidence that NK cells crosstalk with neutrophils and these interactions are bi-directional^14^. In this study, we aimed to analyze whether activated NK cells may synthesize soluble factors which limit NET release or if NK cells can provide suppressive signal to neutrophils directly by cell-to-cell interactions. We isolated NK cells from peripheral blood of healthy volunteers and cultured them with IL-2 or IL-15 (Supplementary Fig. 8a), which provided a signal for NK cells activation and expansion, as confirmed by the formation of cellular aggregates (Supplementary Fig. 8b) and increased expression of NKp30 and/or NKp46 activating receptors (Supplementary Fig. 8c). In general, higher expression of activating receptors was observed in case of IL-2-triggered activation than in IL-15-activated NK cells.

Next, we investigated the influence of soluble factors released by activated NK cells on NET release. We found that NET formation remained unaltered in the presence of NK cells secretome (Supplementary Fig. 9a,b) or activated NK cells (Supplementary Figs. 10, 11). Interestingly, quantitative analyses pointed to a very slight, yet statistically significant (p = 0.0423), increase in the spontaneous formation of NETs in the presence of IL-15 alone in unstimulated samples (Supplementary Fig. 9b). This effect was not observed in the presence of any of NETs-inducing agents. Altogether, NET release seems to be independent from the influence of activated NK cells.

## Discussion

The phenomenon of NET formation was first described almost two decades ago and since then, it drew attention of numerous research groups and has become a widely studied area. It has been proven beyond any doubt that disbalance between NET formation and degradation contributes to the development of multiple diseases, including autoimmune diseases, cancers, thrombotic disorders, and others^22^. NET formation has been put in the spotlight during COVID-19 pandemic, since exacerbated NET release has been shown to participate in molecular mechanisms driving damage of distant organs^29^. Notably, most of the foregoing studies have been undertaken to identify factors driving NET release, but factors limiting NET formation remain largely unknown. NETs can be degraded in vivo by DNases and NETs fragments can be cleared by macrophages^30^, but there is a gap in knowledge regarding agents suppressing the process of NET release on the level of neutrophil activation. Accordingly, the aim of this study was to identify factors limiting NET formation. Here we provide evidence that supernatants derived from M1, M2a and M2c macrophages suppress NET release as well as diminish ROS synthesis. Addition of H_2_O_2_ as an external source of ROS reverted the inhibitory effect of macrophage secretome on NET formation. We failed to show the similar NET-inhibiting properties of Tregs or NK cells secretomes. Direct interactions of neutrophils with macrophages, Tregs or NKs did not affect NET release. Similarly, NET formation was not influenced by cytokines (IL-4, IL-10, TGF-β) or nucleoside of immunosuppressive properties—adenosine.

Immune response, including cellular activation, proliferation and differentiation, is orchestrated by the coordinated interplay between various cytokines. Neutrophils, among other immune cells, can both synthesize cytokines^31^, as well as sense signals provided by cytokine signaling^32^—autocrine, or derived from neighboring and distant cells (paracrine and endocrine signaling)^33^. Notably, cytokines are divided into those of pro-inflammatory and anti-inflammatory activity, but this division is not absolute, since many cytokines can act as pro- or anti-inflammatory agents, depending on the clinical and immunological setting^34^. We aimed to identify factors limiting NET formation, and since pro-inflammatory cytokines, such as tumor necrosis factor (TNF) α, IL-1β and IL-8, have been previously shown to induce NET release^35,36^, we focused on anti-inflammatory cytokines. Previous research showed that cytokines of classically anti-inflammatory activity may regulate neutrophils effector functions. For example, IL-10 enhances phagocytosis of murine granulocytes^37^, IL-4 and IL-13 antagonize neutrophils migration^8,38^, and IL-11 is involved in neutrophil recruitment to infected lungs. Several studies investigated the influence of anti-inflammatory cytokines and other immunosuppressive factors on NET release, providing evidence that IL-4, IL-13, and adenosine diminish PMA-induced NET release^8^. Surprisingly, studies showed NETs-inducing properties of IL-10, nevertheless only up to 9% of neutrophils released NETs in response to stimulation with this cytokine^39^. The literature on the role of another anti-inflammatory cytokine, TGF-β, is not consistent. On one hand it was shown that TGF-β induces NET release^40^, but on the other hand it did not affect PMA-induced NET release or NET release following FcγRIIIb cross-linking^41^. In the light of limited number of NETs inducers used in the aforementioned studies, as well as conflicting reports, there was a need for more comprehensive analyses regarding the influence of anti-inflammatory agents on NET release. We analyzed the influence of multiple concentrations of IL-4, IL-10, TGF-β, and adenosine, stimulating the cells with a wide range of inducers involving various molecular mechanisms, including: PAF, LPS isolated from *E. coli*, PMA, CI, and ONOO^−^. However, we did not observe inhibitory effect of any of these agents on NET release. These results suggest that the influence of various anti-inflammatory cytokines on NET release may be dependent on the type of NETs-inducing agent, the concentrations of cytokine and NETs inducer, as well as the conditions under which NETs studies are conducted.

The dependence between macrophages and NET release has been extensively studied before, and the vast majority of these reports addresses the influence of NETs on cytokine release by macrophages, or the role of macrophages in NETs degradation. In general, these studies provide evidence that NETs-treated macrophages release increased amounts of cytokines, mostly of pro-inflammatory activity^42–47^, and identify macrophages as key cells engulfing NETs fragments^48–50^. Additionally, it was demonstrated that the engulfment of NETs containing neutrophil-specific antimicrobial peptides by macrophages augments macrophage killing of bacteria^51^. On the other hand, the influence of neutrophil-macrophage interactions on NET release remains an obscure area of studies. To date, research has provided evidence that aberrant expression of macrophage-derived microRNAs miR-4512 and miR-146a contributes to exaggerated NET formation in patients suffering from SLE and atherosclerosis, respectively^17,52^. Furthermore, bovine neutrophils have been shown to release less NETs when in direct contact with macrophages, which allowed for reduced inflammatory reaction and enhanced killing of *Mycobacterium bovis* Bacillus-Calmette-Guérin (BCG)^16^. In line with this, we have observed that macrophages can inhibit NET formation, but in humans this effect was driven by soluble factors present in macrophages-derived supernatants. Notably, the inhibition of NET release by macrophage supernatants was observed for each NETs inducer used in our studies, i.e. PAF, ONOO^-^, PMA and LPS *E. coli*, with the effect least pronounced in the presence of PMA. This suggests that macrophage secretome either affects a molecular mechanism shared by various NETs inducers, or macrophage secretome constituents exhibit pleiotropic effects on molecular events underlying NET formation. The inhibitory effect of M1, M2a and M2c macrophage secretome on NET release was not prevented by boiling of the supernatants, which suggests that it is driven by heat-stable molecule(s): lipid(s), oligosaccharide(s) or small peptide(s) capable of refolding after denaturation, but not proteins such as cytokines or chemokines^53^. M2a macrophages synthesize higher amounts or more efficient factor(s) inhibiting NET release than M1 and M2c macrophages, as suggested by the results of supernatants dilution experiments.

Notably, we observed the inhibition of NET release by macrophage secretome, but the lack of this inhibition in neutrophil-macrophage co-culture experiments. It might raise concerns about the biological relevancy of this observation, since it is reasonable to assume that polarized macrophages directly contacting neutrophils should still synthesize factor(s) limiting NET formation and be able to diminish NET release. We believe that the amount of factor(s) synthesized by macrophages during the course of 1-h co-culture experiment is lower than during 24-h polarization and thus, may not be high enough to inhibit NET formation. However, it does not exclude that in vivo, when various immune cells infiltrate the site of infection or inflammation and remain functional for multiple days, macrophages can synthesize high amounts of molecule(s) affecting the function of neighboring neutrophils and thus inhibit NET release.

One could argue that diminished NET formation in the presence of macrophage supernatant is the result of NETs degradation and not inhibition of neutrophil activation. However, microscopical analyses pointed to the presence of increased amounts of cells at the early stages of NET formation (i.e. polymorphonuclear neutrophils or decondensed neutrophil with non-permeabilized plasma membrane) in the presence of macrophage supernatant. This observation confirms that macrophage supernatant is responsible for the inhibition of NET formation at the early stages of the process.

We also aimed to elucidate whether this inhibitory effect of macrophage supernatants on NET release can be attributed to their ROS-limiting properties. We provided evidence that diminished ROS synthesis by M1 macrophage supernatant could at least partially explain its suppressive effect on NET release. Interestingly, the inhibitory effect of M2 macrophage secretome on oxidative burst of neutrophils was only observed for M2a macrophages and only when CI was used as an inducer. IL-10 alone inhibited CI-induced oxidative burst of neutrophils and in the presence of M2c macrophages supernatant, containing IL-10, this effect was not further enhanced. These experiments pointed to various effects of M1, M2a and M2c macrophages supernatants on neutrophils functions.

Previous studies pointed to various sources of ROS during NET formation with either NADPH oxidase-derived ROS or mitochondrial ROS playing the dominant role^26,54^. Our experiments highlighted that macrophage supernatant does not affect the synthesis of mitochondrial superoxide, suggesting that the inhibition of NADPH oxidase by macrophage secretome may underlie the observed phenomenon.

Inhibitory effect of macrophage secretome on NET release was abrogated by the external source of ROS, H_2_O_2_. This observation further implies that ROS synthesis might be a major NET-related mechanism affected by soluble factor(s) derived from polarized macrophages. Nonetheless, it cannot be excluded that macrophage secretome affects not only oxidative burst, but also activity of other processes and molecules critical for NET release, such as histone citrullination, proteolytic cleavage of histones by NE, or activation of protein kinases^22^. Future studies are warranted to identify factors and detailed mechanisms responsible for the observed phenomenon.

Regulatory T cells, identified by the expression of CD4, CD25, and FOXP3 has been repeatedly pointed as key players in the regulation of the immune system by the maintenance of immune tolerance and homeostasis. Recently there is a significant interest in the impact of Tregs on the cells of the innate immune system^55^. It has been shown that Tregs can affect neutrophils functions by cell–cell contact mechanism and the secretion of cytokines. Richards et al. showed that Tregs can influence acute inflammation in the skin by suppressing neutrophil accumulation and survival, whereas Lewkowicz et al. revealed that LPS-stimulated Tregs can inhibit reactive oxygen intermediates and cytokine production as well as accelerate neutrophil apoptosis^56,57^. Further studies by Lewkowicz et al. showed that activated Treg cells promote generation of IL-10 and TGF-β by neutrophils and induce the expression of heme oxygenase-1 (HO-1), indoleamine 2,3-dioxygenase (IDO) and the suppressor of cytokine signaling 3 molecule (SOCS3)^58^. These studies on the interactions between neutrophils and Tregs underline the role of Tregs in the regulation of innate immune responses through the induction of immunosuppressive properties of neutrophils. However, in our experimental settings neither supernatant from Tregs nor IL-2, present in the culture and supernatants, inhibited NET release. Similarly, we did not observe any impact of Tregs on NET formation in samples containing neutrophils co-cultured with Tregs. Our findings suggest that previously described immunosuppressive properties of Tregs are not related to the phenomenon of NET formation.

NK cells constitute an important component of the innate immune system. NK cells mediate spontaneous “natural” cytotoxicity to tumor- and virus-infected cells and are the major source of TNF-α, GM-CSF, IFN-γ, as well as other cytokines and chemokines^59^. Moreover, NK cells are involved in cross-talk with monocytes, macrophages and, according to the latest reports, with neutrophils. NK cells may affect neutrophils survival, recruitment, and functional responses^14^. Costantini et al. showed that NK cells activated with various cytokines, including IL-2, IL-15, IL-18 or IL-21, prolonged the survival of neutrophils via the release of IFN-γ, GM-CSF, or other unidentified soluble factor(s)^60^. It was also reported that NK cells exposed to IL-2 and IL-15 may up-regulate expression of neutrophil’s activation markers such as CD64 and CD69^60,61^. Furthermore, supernatants harvested from NK cells activated with IL-2 modified phagocytic activity and neutrophil’s ROS production^60^. What is more, Bertin et al. demonstrated that IFN- γ produced by NK cells mediated NET formation in murine model of deep vein thrombosis^15^. In contrast, our study has shown that NET formation in humans remained unaltered in the presence of NK cells secretome or activated NK cells.

In this novel study, we were able to identify NET-regulating properties of macrophage secretome. A notable strength of our approach is the use of a wide range of NET inducers, employing various mechanisms of NET release^62^, which validates our observations. Another strength of our study was using monocyte-derived macrophages (MDM) as a source of secretome, and not differentiated cell lines, e.g. THP-1, since MDM functionally better represent the behavior of human macrophages^63^. In our studies we analyzed the effects of secretome collected from unpolarized, as well as differentially polarized macrophages (types M1 and M2 with further subtypes M2a and M2c), which enables comparison of functionally divergent populations. Lastly, sample sizes were large enough to yield statistically significant results. On the other side, our findings have to be seen in light of some limitations. A major source of limitation in this study is utilizing in vitro approach. Although it is widely accepted in biomedicine, naturally it is not as complex as in vivo setting^64^. It cannot be excluded that the very same cytokines and secretomes of NK cells and Tregs, which we analyzed in vitro without observing any apparent effect on neutrophils functions, may in fact very efficiently regulate NET formation inside living organisms. It is possible that various molecules secreted by tissues surrounding neutrophils, factors secreted by endothelium, circulating cytokines, interaction with adhesion molecules etc., sensitize neutrophils and facilitate the response to anti-inflammatory agents. Accordingly, the presented study is just the first step to elucidate mechanisms limiting NET formation and should be complemented in the future by studies utilizing more advanced, complex models, e.g. 3D cultures, or in vivo approach. Furthermore, it cannot be excluded that NET release would have been influenced, if other ratios of neutrophils to macrophages/NK cells or Tregs had been used in this study. It is plausible that under inflammatory conditions, other ratios of immune cells than used in this study (based on^58,60,65–67^) are found in inflamed areas, affecting the final outcome of intercellular interactions and further studies are warranted. Lastly, in our study we only analyzed the influence of macrophage secretome on ROS synthesis, but it needs to be highlighted that other NET-related molecular pathways and mechanisms, such as histone citrullination and cleavage or activation of various protein kinases, may also be affected by macrophage-derived factor(s).

Overall, our study highlights the importance of paracrine signaling between various immune cells at the site of infection and is the first to show that factors released by macrophages can negatively regulate NET formation. On the contrary, similar activity could not be attributed to secretomes of activated NK cells or Tregs. NET synthesis seems to be independent of anti-inflammatory agents, such as TGF-β, IL-4, IL-10, as well as direct cell–cell interactions between neutrophils and NK cells, macrophages or Tregs. This study provides an important insight into factors limiting exacerbated NET formation at the site of infection and inflammation, with macrophage-derived soluble mediators as major contributors. Further studies are warranted to identify specific molecules and mechanisms responsible for the observed phenomenon.

## Materials and methods

### Reagents

Roswell Park Memorial Institute (RPMI) 1640 medium, antibiotic–antimycotic solution, HEPES buffer, micrococcal nuclease (MNase), SYTOX Green, MitoSOX Red, SYTOX Orange and Hoechst 33,342 were purchased from Thermo Fisher Scientific (Waltham, Massachusetts, USA). Platelet activating factor C-16 (PAF), PMA, and sodium peroxynitrite were purchased from Cayman (Ann Arbor, MI, USA). Fetal bovine serum (FBS) was purchased from Biochrom (Berlin, Germany). H_2_O_2_ was purchased from POCH (Gliwice, Poland). GM-CSF, M-CSF, IL-2, IL-4, IL-10, IL-15, TGF-β2, IFN-γ were purchased from PeproTech (Cranbury, New Jersey, United States). IL-2 was purchased from STEMCELL Technologies (Vancouver, Canada). LPS isolated from *E. coli*, adenosine, DHR 123, DNAse, PMA and all other reagents, unless otherwise stated, were purchased from Sigma Aldrich (St Louis, MO, USA).

### Isolation of neutrophils, monocytes, NK cells, and regulatory T cells

To isolate human immune cells, blood samples or buffy coats from healthy blood donors were purchased at the Regional Blood Donation Center. Alternatively, blood was collected from healthy volunteers, without any symptoms of current infection, after giving an informed consent. This study was approved by the Institutional Ethics Committee of Medical University of Warsaw (KB/22/2020).

Since NK cells and T cells recognize self from non-self antigens, neutrophils and NK cells or Tregs for co-culture experiments were isolated from the same human subjects^68^. Macrophages and neutrophils used for co-culture experiments were isolated from various human subjects.

Neutrophils were isolated by density gradient centrifugation and by polyvinyl sedimentation method as described previously^69^. Monocytes, Tregs and NK cells were isolated by immunomagnetic methods with the use of commercial kits purchased from STEMCELL Technologies and isolation procedure was performed as described in manufacturer’s protocols. Monocytes were isolated with the EasySep™ Human Monocyte Enrichment Kit without CD16 Depletion, Tregs were isolated with EasySep™ Human CD4+ CD127lowCD25+ Regulatory T Cell Isolation Kit and NK cells were isolated with EasySep™ Human NK Cell Isolation Kit.

### Differentiation of monocytes, collection of supernatant

The overview of the protocol used for monocyte differentiation and supernatant collection is shown in Supplementary Fig. 2a. The protocols used for monocyte culture and differentiation were based on the procedure published by Kelly et al.^70^.

Isolated monocytes were suspended in RPMI-1640 medium supplemented with 10% FBS and antibiotic–antimycotic solution (further referred to as “full RPMI medium”) and seeded into the wells of appropriate dishes: LabTek chambers for immunostaining or multi-well plastic plates with flat bottom for live imaging at the density of 2.5 × 10^4^–5 × 10^5^ cells/ml. Medium was supplemented with 50 ng/ml GM-CSF to differentiate monocytes into M1 (proinflammatory) macrophages, or with 50 ng/ml M-CSF to differentiate monocytes into M2 (anti-inflammatory) macrophages. The medium was partially replaced and fresh cytokines were added on the 3rd or 4th day of differentiation. After 6 days of differentiation, the medium was completely removed and replaced with full RPMI containing 50 ng/ml interferon gamma (IFN-γ; M1 macrophages), 20 ng/ml interleukin 4 (IL-4; M2a macrophages) or 20 ng/ml IL-10 (M2c macrophages). The cells were incubated for the following 24 h and on the 7th day of differentiation the cells were used for experiments.

To confirm the differentiation of monocytes into monocyte-derived macrophages of pro- or anti-inflammatory phenotypes, macrophages were harvested with the use of a cell scraper and stained with the following cocktail of antibodies: anti-CD14 (555399, BD Pharmingen; BD; Franklin Lakes, NJ, USA; 20 μl/10^6^ cells), anti-CD16 (560195, BD Pharmingen, 5 μl/10^6^ cells), anti-CD45 (560777, BD Horizon, 5 μl/10^6^ cells), anti-CD68 (562117, BD Pharmingen, 5 μl/10^6^ cells), anti-CD80 (564160, BD Horizon, 5 μl/10^6^ cells), anti-CD163 (BD Pharmingen, 20 μl/10^6^ cells). The samples were analyzed with the use of BD LSRFortessa flow cytometer.

For co-culture experiments, on the 7th day of differentiation, full medium in the wells containing macrophages was replaced with serum-free RPMI-1640 medium with the addition of 10 mM HEPES buffer, further referred to as RH medium. Subsequently, neutrophils were seeded into the wells containing M1 or M2 macrophages at the ratio of 2 neutrophils: 1 macrophage. NET formation was stimulated as described below.

To harvest supernatant, RH medium, instead of full RPMI, containing IFN-γ, IL-4 or IL-10 was added to the macrophages on the 6th day of differentiation (1 ml of medium per 2.5 × 10^5^ cells). After 24 h, the supernatants were collected, filtered through a 0.2–0.45 μm PVDF filter and kept at − 80 °C until further use. In some experiments, supernatant was boiled (95 °C, 10 min) and then cooled to room temperature prior to addition to the cells.

### Activation of regulatory T cells, collection of supernatant

The overview of the protocol used for Tregs activation and supernatant collection is shown in Supplementary Fig. 5a.

Isolated Tregs were suspended in full RPMI medium and seeded into multi-well plastic plates with flat bottom at the density of 2 × 10^5^ cells/ml. Medium was supplemented with 100 IU/ml IL-2 and 25 μl/ml of ImmunoCult™ Human CD3/CD28/CD2 T Cell Activator (STEMCELL Technologies) was added to the cell suspension. The cells were incubated for 2 days. Activated Tregs were washed with saline before being used for experiments.

To confirm the activation and expansion of Tregs, the cells were visualized with the use of inverted microscope Leica DMi8 and activation of the cells was assessed as the presence of Tregs conglomerates. Additionally, the expression of CD69, an early marker of Tregs activation, was analyzed with BD LSRFortessa flow cytometer after the staining with Human Regulatory T Cell Cocktail (560249, BD Pharmingen) and anti-CD69 antibody (562884, BD Horizon) (5 μl of each reagent per test, i.e. 2 × 10^4^ cells).

For co-culture experiments, activated Tregs were resuspended in RH medium and seeded into LabTek chambers at the density of 5 × 10^4^ cells/ml. Subsequently, neutrophils were seeded into the wells containing Tregs at the ratio of 10 neutrophils: 1 Treg and NET formation was stimulated as described below.

To harvest supernatant, activated Tregs were resuspended in RH medium at the density of 2 × 10^5^ cells/ml and cultured in the presence of IL-2 for 24 h. The cells were then centrifuged, supernatants were collected, filtered through a 0.45 μm PVDF filter and kept at − 80 °C until further use.

### Activation of NK cells, collection of supernatant

The overview of the protocol used for NK cells activation and supernatant collection is shown in Supplementary Fig. 8a.

Isolated NK cells were suspended in full RPMI medium and seeded into multi-well plastic plates with flat bottom at the density of 5 × 10^5^ cells/ml^71^. To activate NK cells, medium was supplemented with 100 IU/ml IL-2 or 10 IU/ml IL-15 and the cells were cultured for 3 days. Activated NK cells were washed with phosphate-buffered saline or saline before being used for experiments.

To confirm the activation and expansion of NK cells, the cells were visualized with the use of inverted microscope Leica DMi8 and activation of the cells was assessed as the presence of cells’ conglomerates. Additionally, the activation of NK cells was assessed by flow cytometry with BD LSRFortessa. To that end, the cells were stained with anti-CD3 antibody (341111, BD Biosciences, 12.5 μl/10^6^ cells), anti-CD45 antibody (560777, BD Horizon, 5 μl/10^6^ cells), anti-CD56 antibody (345811, BD Biosciences, 50 μl/10^6^ cells), anti-CD16 antibody (560195, BD Pharmingen, 5 μl/10^6^ cells), anti-CD337 antibody (anti-NKp30, 563385, BD Horizon, 5 μl/10^6^ cells), and anti-CD335 antibody (anti-NKp46, 557991, BD Pharmingen, 20 μl/10^6^ cells).

For co-culture experiments, activated NK cells were resuspended in RH medium and seeded into LabTek chambers at the density of 3.75 × 10^4^ cells/ml. Neutrophils were seeded into the wells containing NK cells at the ratio of 1 neutrophils: 1 NK cell and NET formation was stimulated as described below.

To harvest supernatant, activated NK cells were resuspended in RH medium and cultured at the density of 5 × 10^5^ cells/ml in the presence of 100 IU/ml IL-2 or 10 IU/ml IL-15. After 24 h, the cells were centrifuged, supernatants were collected, filtered through a 0.45 μm PVDF filter and kept at − 80 °C until further use.

### Neutrophil extracellular traps formation assay: stimulation, visualization and quantification

To induce NET release, neutrophils were suspended in RH medium and seeded at 2–2.5 × 10^4^ cells per well into 8-well Lab-Tek chambers for NETs immunostaining; 5 × 10^4^ cells per well were seeded into 24-well plates for extracellular DNA quantification; 1–2 × 10^4^ cells per well were seeded into 48- or 96-well plates for live NETs imaging. Subsequently, increasing concentration of IL-4, IL-10, TGF-β2 or adenosine were added to the wells and neutrophils were incubated for 1 h at 37 °C, 5% CO_2_. When the influence of various supernatants on NET release was studied, supernatant was added to the wells in such an amount so that it constituted 50% of final volume, and the cells were incubated for 1 h. When indicated, supernatants were diluted 1:2, 1:5, and 1:10 with RH medium prior to addition to the wells. Alternatively, neutrophils were co-cultured with macrophages, Tregs or NK cells at the ratios indicated above for 1–2 h, as described in Figure legends. Subsequently, neutrophils were stimulated with: 100 µM peroxynitrite, 2.5 µM PAF, 5 µg/ml LPS isolated from *E. coli*, 4 μM CI or 100 nM PMA. When the influence of exogenous source of ROS on NET release was studied, 30 μM H_2_O_2_ was added to the cells simultaneously with an inducer of NET release. Unstimulated cells served as control. Samples were incubated for the following 3 h and then NET formation was analyzed.

To visualize NETs in unfixed samples (live cell imaging), samples were stained with 1.25 µg/ml Hoechst 33342 and 100 nM SYTOX Green. Accordingly, total DNA was visualized with blue color and extracellular DNA (including NETs) was visualized with green color. Samples were visualized at the objective magnification 10 × with Leica DMi8 microscope (Leica, Wetzlar, Germany). To quantify NET release, at least two images per each sample were taken and the percentage coverage of image area by NETs was assessed with the use of Part Seg software v. 0.13.14^72^ with Trapalyzer Plugin as described in Ref.^73^.

Alternatively, to quantify NET release, the amount of extracellular DNA released by neutrophils was measured fluorometrically. To that end, 10 U/ml DNAse or 500 mIU/ml MNase was added to the wells to detach DNA, and the samples were incubated for 10 or 20 min, respectively, at 37 °C. The samples were further processed exactly as described in Ref.^23^.

Besides live NETs imaging, NETs were also visualized following immunostaining. The samples were fixed with 4% paraformaldehyde (PFA) for 20 min, permeabilized with 0.1% Triton X, blocked with 1% BSA for 30 min, and then neutrophils were stained with anti-myeloperoxidase antibodies (ab11729, Abcam, Cambridge, UK; 1:500, overnight, 4 °C), and DNA was counterstained with 1 µM SYTOX Orange. When the influence of macrophages on NET release was studied, the samples were fixed with PFA, permeabilized with ice-cold methanol for 10 min, and blocked with 5% goat serum for 60 min. The macrophages were stained with anti-CD68 antibodies (#76437; Cell Signaling, Beverly, Massachusetts, USA; 1:800) overnight at 4 °C. Subsequently, samples were incubated with secondary antibody (1:1000, ab6717, 1 h, room temperature) and DNA was counterstained with SYTOX Orange. Slides were assessed with Leica DMi8 fluorescent microscope equipped with a 40 × and a 10 × magnification objectives.

### Reactive oxygen species measurement

Neutrophils were seeded into the wells of black 96-well plates at the density of 5 × 10^5^/ml. Supernatants collected from M1 or M2 macrophages were added to the wells so that they constituted 50% of the final volume. Subsequently, the cells were loaded with DHR. After 30 min of incubation at 37 °C, 5% CO_2_, the cells were washed to remove excess probe, fresh supernatants were added and neutrophils were stimulated with 100 nM PMA, 100 μM H_2_O_2_ or 4 μM CI. ROS synthesis was monitored fluorometrically every 15 min throughout 4 h post stimulation with the use of FLUOstar OMEGA plate reader (BMG Labtech, Ortenberg, Germany).

To measure the synthesis of mitochondrial ROS, 5 × 10^5^ cells suspended in 250 μl RH were incubated for 1 h with 250 μl of supernatants collected from M1 or M2 macrophages. After 1-h incubation, the cells were stimulated with 100 nM PMA, incubated for 1 h and then washed with saline and loaded with 5 μM MitoSOX for 30 min. All incubation steps were performed at 37 °C, 5% CO_2_. Subsequently, the samples were washed with saline and analyzed with BD LSRFortessa flow cytometer.

### Statistical analyses

Data analysis was performed with GraphPad Prism Software v. 9 (GraphPad Software, La Jolla, CA, USA). Shapiro–Wilk normality test was routinely used to check normality of data. All tests were two-tailed. Multiple groups were compared with one-way ANOVA or Friedman test with appropriate post-hoc tests, unless specified otherwise. P ≤ 0.05 was considered statistically significant. Whenever the number of individual experiments (n) is specified in Figure legends, it refers to biological replicates.

### Ethics approval

The study was conducted in accordance with the Declaration of Helsinki, and approved by the Institutional Ethics Committee of Medical University of Warsaw (KB/22/2020, date of approval: 03 Feb 2020).

### Consent to participate

For this study, blood was either purchased at local blood donation center or collected from healthy volunteers. When the blood was collected from healthy volunteers, an informed, written consent was signed by each individual. According to local law, blood donors gave the blood donation center a written permission to sell their blood samples/constituents for scientific purposes so an additional, written informed consent was not necessary.

### Supplementary Information

Supplementary Figures.

## Data Availability

The data presented in this study are available on request from the corresponding author.

## References

[1] Brinkmann V (2004). Neutrophil extracellular traps kill bacteria. Science.

[2] Kaplan MJ, Radic M (2012). Neutrophil extracellular traps: Double-edged swords of innate immunity. J. Immunol..

[3] Merza M (2015). Neutrophil extracellular traps induce trypsin activation, inflammation, and tissue damage in mice with severe acute pancreatitis. Gastroenterology.

[4] Darrah E, Andrade F (2013). NETs: the missing link between cell death and systemic autoimmune diseases?. Front. Immunol..

[5] Cools-Lartigue J (2013). Neutrophil extracellular traps sequester circulating tumor cells and promote metastasis. J. Clin. Investig..

[6] Zawrotniak M, Rapala-Kozik M (2013). Neutrophil extracellular traps (NETs)—Formation and implications. Acta Biochim. Pol..

[7] Xu K (2019). Adenosine from a biologic source regulates neutrophil extracellular traps (NETs). J. Leukoc. Biol..

[8] Impellizzieri D (2019). IL-4 receptor engagement in human neutrophils impairs their migration and extracellular trap formation. J. Allergy Clin. Immunol..

[9] Yang F, Feng C, Zhang X, Lu J, Zhao Y (2017). The diverse biological functions of neutrophils, beyond the defense against infections. Inflammation.

[10] Mocsai A (2013). Diverse novel functions of neutrophils in immunity, inflammation, and beyond. J. Exp. Med..

[11] Mantovani A, Cassatella MA, Costantini C, Jaillon S (2011). Neutrophils in the activation and regulation of innate and adaptive immunity. Nat. Rev. Immunol..

[12] Lewkowicz N (2016). Induction of human IL-10-producing neutrophils by LPS-stimulated Treg cells and IL-10. Mucosal Immunol..

[13] Daseke M, Kalusche W, Flynn E, Konfrst S, Lindsey M (2020). Infarct macrophage secretome coordinates neutrophil degranulation. FASEB J..

[14] Costantini C, Cassatella MA (2011). The defensive alliance between neutrophils and NK cells as a novel arm of innate immunity. J. Leukoc. Biol..

[15] Bertin FR (2019). Natural killer cells induce neutrophil extracellular trap formation in venous thrombosis. J. Thromb. Haemost..

[16] Ladero-Aunon I (2021). Bovine neutrophils release extracellular traps and cooperate with macrophages in *Mycobacterium avium* subsp. paratuberculosis clearance in vitro. Front. Immunol..

[17] Yang B (2021). Decreased miR-4512 levels in monocytes and macrophages of individuals with systemic lupus erythematosus contribute to innate immune activation and neutrsophil NETosis by targeting TLR4 and CXCL2. Front. Immunol..

[18] Marie C, Pitton C, Fitting C, Cavaillon JM (1996). Regulation by anti-inflammatory cytokines (IL-4, IL-10, IL-13, TGFbeta)of interleukin-8 production by LPS- and/or TNFalpha-activated human polymorphonuclear cells. Mediat. Inflamm..

[19] Opal SM, DePalo VA (2000). Anti-inflammatory cytokines. Chest.

[20] da Rocha Lapa F, da Silva MD, de Almeida Cabrini D, Santos AR (2012). Anti-inflammatory effects of purine nucleosides, adenosine and inosine, in a mouse model of pleurisy: Evidence for the role of adenosine A2 receptors. Purinergic Signal..

[21] Hasko G, Cronstein B (2013). Regulation of inflammation by adenosine. Front. Immunol..

[22] Papayannopoulos V (2018). Neutrophil extracellular traps in immunity and disease. Nat. Rev. Immunol..

[23] Manda-Handzlik A (2020). Nitric oxide and peroxynitrite trigger and enhance release of neutrophil extracellular traps. Cell. Mol. Life Sci..

[24] Huang X, Li Y, Fu M, Xin HB (2018). Polarizing macrophages in vitro. Methods Mol. Biol..

[25] Prame Kumar K, Nicholls AJ, Wong CHY (2018). Partners in crime: Neutrophils and monocytes/macrophages in inflammation and disease. Cell Tissue Res..

[26] Douda DN, Khan MA, Grasemann H, Palaniyar N (2015). SK3 channel and mitochondrial ROS mediate NADPH oxidase-independent NETosis induced by calcium influx. Proc. Natl. Acad. Sci. U.S.A..

[27] Neubert E (2019). Serum and serum albumin inhibit in vitro formation of neutrophil extracellular traps (NETs). Front. Immunol..

[28] Reddy M, Eirikis E, Davis C, Davis HM, Prabhakar U (2004). Comparative analysis of lymphocyte activation marker expression and cytokine secretion profile in stimulated human peripheral blood mononuclear cell cultures: an in vitro model to monitor cellular immune function. J. Immunol. Methods.

[29] Ackermann M (2021). Patients with COVID-19: In the dark-NETs of neutrophils. Cell Death Differ..

[30] Farrera C, Fadeel B (2013). Macrophage clearance of neutrophil extracellular traps is a silent process. J. Immunol..

[31] Tecchio C, Micheletti A, Cassatella MA (2014). Neutrophil-derived cytokines: Facts beyond expression. Front. Immunol..

[32] Glennon-Alty L, Moots RJ, Edwards SW, Wright HL (2021). Type I interferon regulates cytokine-delayed neutrophil apoptosis, reactive oxygen species production and chemokine expression. Clin. Exp. Immunol..

[33] Papanicolaou DA, Vgontzas AN (2000). Interleukin-6: The endocrine cytokine. J. Clin. Endocrinol. Metab..

[34] Bordon J (2013). Understanding the roles of cytokines and neutrophil activity and neutrophil apoptosis in the protective versus deleterious inflammatory response in pneumonia. Int. J. Infect. Dis..

[35] Keshari RS (2012). Cytokines induced neutrophil extracellular traps formation: Implication for the inflammatory disease condition. PLoS ONE.

[36] Meher AK (2018). Novel role of IL (interleukin)-1 beta in neutrophil extracellular trap formation and abdominal aortic aneurysms. Arterioscler. Thromb. Vasc. Biol..

[37] Mittal R (2010). IL-10 administration reduces PGE-2 levels and promotes CR3-mediated clearance of *Escherichia coli* K1 by phagocytes in meningitis. J. Exp. Med..

[38] Heeb LEM, Egholm C, Boyman O (2020). Evolution and function of interleukin-4 receptor signaling in adaptive immunity and neutrophils. Genes Immun..

[39] Garley M (2017). Cytokine network & NETs. Folia Biol. (Praha).

[40] Jablonska E (2020). Neutrophil extracellular traps (NETs) formation induced by TGF-beta in oral lichen planus—Possible implications for the development of oral cancer. Immunobiology.

[41] Aleman OR, Mora N, Cortes-Vieyra R, Uribe-Querol E, Rosales C (2016). Transforming growth factor-beta-activated kinase 1 is required for human fc gamma riiib-induced neutrophil extracellular trap formation. Front. Immunol..

[42] Chen X, Li Y, Qin L, He R, Hu C (2021). Neutrophil extracellular trapping network promotes the pathogenesis of neutrophil-associated asthma through macrophages. Immunol. Investig..

[43] An Z (2019). Neutrophil extracellular traps induced by IL-8 aggravate atherosclerosis via activation NF-kappaB signaling in macrophages. Cell Cycle.

[44] Warnatsch A, Ioannou M, Wang Q, Papayannopoulos V (2015). Neutrophil extracellular traps license macrophages for cytokine production in atherosclerosis. Science.

[45] Josefs T (2020). Neutrophil extracellular traps promote macrophage inflammation and impair atherosclerosis resolution in diabetic mice. JCI Insight.

[46] Eghbalzadeh K (2019). Compromised anti-inflammatory action of neutrophil extracellular traps in PAD4-deficient mice contributes to aggravated acute inflammation after myocardial infarction. Front. Immunol..

[47] Apel F (2021). The cytosolic DNA sensor cGAS recognizes neutrophil extracellular traps. Sci. Signal..

[48] Gregoire M (2018). Impaired efferocytosis and neutrophil extracellular trap clearance by macrophages in ARDS. Eur. Respir. J..

[49] Jeong JH (2021). Neutrophil extracellular trap clearance by synovial macrophages in gout. Arthritis Res. Ther..

[50] Nakazawa D (2016). The responses of macrophages in interaction with neutrophils that undergo NETosis. J. Autoimmun..

[51] Monteith AJ, Miller JM, Maxwell CN, Chazin WJ, Skaar EP (2021). Neutrophil extracellular traps enhance macrophage killing of bacterial pathogens. Sci. Adv..

[52] Zhang YG (2019). Exosomes derived from oxLDL-stimulated macrophages induce neutrophil extracellular traps to drive atherosclerosis. Cell Cycle.

[53] O'Connor JC, Farach-Carson MC, Schneider CJ, Carson DD (2007). Coculture with prostate cancer cells alters endoglin expression and attenuates transforming growth factor-beta signaling in reactive bone marrow stromal cells. Mol. Cancer Res..

[54] Fuchs TA (2007). Novel cell death program leads to neutrophil extracellular traps. J. Cell Biol..

[55] Okeke EB, Uzonna JE (2019). The pivotal role of regulatory T cells in the regulation of innate immune cells. Front. Immunol..

[56] Lewkowicz P, Lewkowicz N, Sasiak A, Tchorzewski H (2006). Lipopolysaccharide-activated CD4(+)CD25(+) T regulatory cells inhibit neutrophil function and promote their apoptosis and death. J. Immunol..

[57] Richards H (2010). Novel role of regulatory T cells in limiting early neutrophil responses in skin. Immunology.

[58] Lewkowicz N, Klink M, Mycko MP, Lewkowicz P (2013). Neutrophil–CD4+CD25+ T regulatory cell interactions: A possible new mechanism of infectious tolerance. Immunobiology.

[59] Campbell KS, Hasegawa J (2013). Natural killer cell biology: An update and future directions. J. Allergy Clin. Immunol..

[60] Costantini C (2010). Neutrophil activation and survival are modulated by interaction with NK cells. Int. Immunol..

[61] Bhatnagar N (2010). Cytokine-activated NK cells inhibit PMN apoptosis and preserve their functional capacity. Blood.

[62] Boeltz S (2019). To NET or not to NET: Current opinions and state of the science regarding the formation of neutrophil extracellular traps. Cell Death Differ..

[63] Tedesco S (2018). Convenience versus biological significance: Are PMA-differentiated THP-1 cells a reliable substitute for blood-derived macrophages when studying in vitro polarization?. Front. Pharmacol..

[64] Jansen A (2022). Ex vivo and in vitro monocyte responses do not reflect in vivo immune responses and tolerance. J. Innate Immun..

[65] Jones CN (2012). Microfluidic chambers for monitoring leukocyte trafficking and humanized nano-proresolving medicines interactions. Proc. Natl. Acad. Sci. U.S.A..

[66] Gray RD (2018). Delayed neutrophil apoptosis enhances NET formation in cystic fibrosis. Thorax.

[67] de Graauw E (2018). Monocytes enhance neutrophil-induced blister formation in an ex vivo model of bullous pemphigoid. Allergy.

[68] Hudson LE, Allen RL (2016). Leukocyte Ig-like receptors—A model for MHC class I disease associations. Front. Immunol..

[69] Bystrzycka W (2016). The effect of clindamycin and amoxicillin on neutrophil extracellular trap (NET) release. Cent. Eur. J. Immunol..

[70] Kelly A, Grabiec AM, Travis MA (2018). Culture of human monocyte-derived macrophages. Methods Mol. Biol..

[71] Fehniger TA (1999). Differential cytokine and chemokine gene expression by human NK cells following activation with IL-18 or IL-15 in combination with IL-12: Implications for the innate immune response. J. Immunol..

[72] Bokota G (2021). PartSeg: A tool for quantitative feature extraction from 3D microscopy images for dummies. BMC Bioinform..

[73] Ciach MA (2023). Trapalyzer: A computer program for quantitative analyses in fluorescent live-imaging studies of neutrophil extracellular trap formation. Front. Immunol..

