# Supplementary material for: Secretomes of M1 and M2 macrophages decrease the release of neutrophil extracellular traps
Source: Sci Rep. 2023 Sep 20;13:15633. doi: 10.1038/s41598-023-42167-1 (PMC10511515; doi:10.1038/s41598-023-42167-1)
Supplement: Supplementary file 1 — Supplementary Figures. [file 41598_2023_42167_MOESM1_ESM.pdf]

Supplementary information

Secretomes of M1 and M2 macrophages decrease the release of neutrophil extracellular traps

Aneta Manda-Handzlik[1*], Adrianna Cieloch[1,2], Weronika Kuźmicka[1], Agnieszka Mroczek[1,2], Anna Stelmaszczyk-Emmel[1], Urszula Demkow[1] and Małgorzata Wachowska[1*]

1 Department of Laboratory Diagnostics and Clinical Immunology of Developmental Age,
Medical University of Warsaw, Zwirki i Wigury 63a Street, 02-091 Warsaw, Poland
2 Doctoral School, Medical University of Warsaw, Zwirki i Wigury 61 Street, 02-091 Warsaw, Poland

Corresponding author:
Aneta Manda-Handzlik, e-mail: aneta.manda-handzlik@wum.edu.pl; telephone number: + 48 22 317 95 03

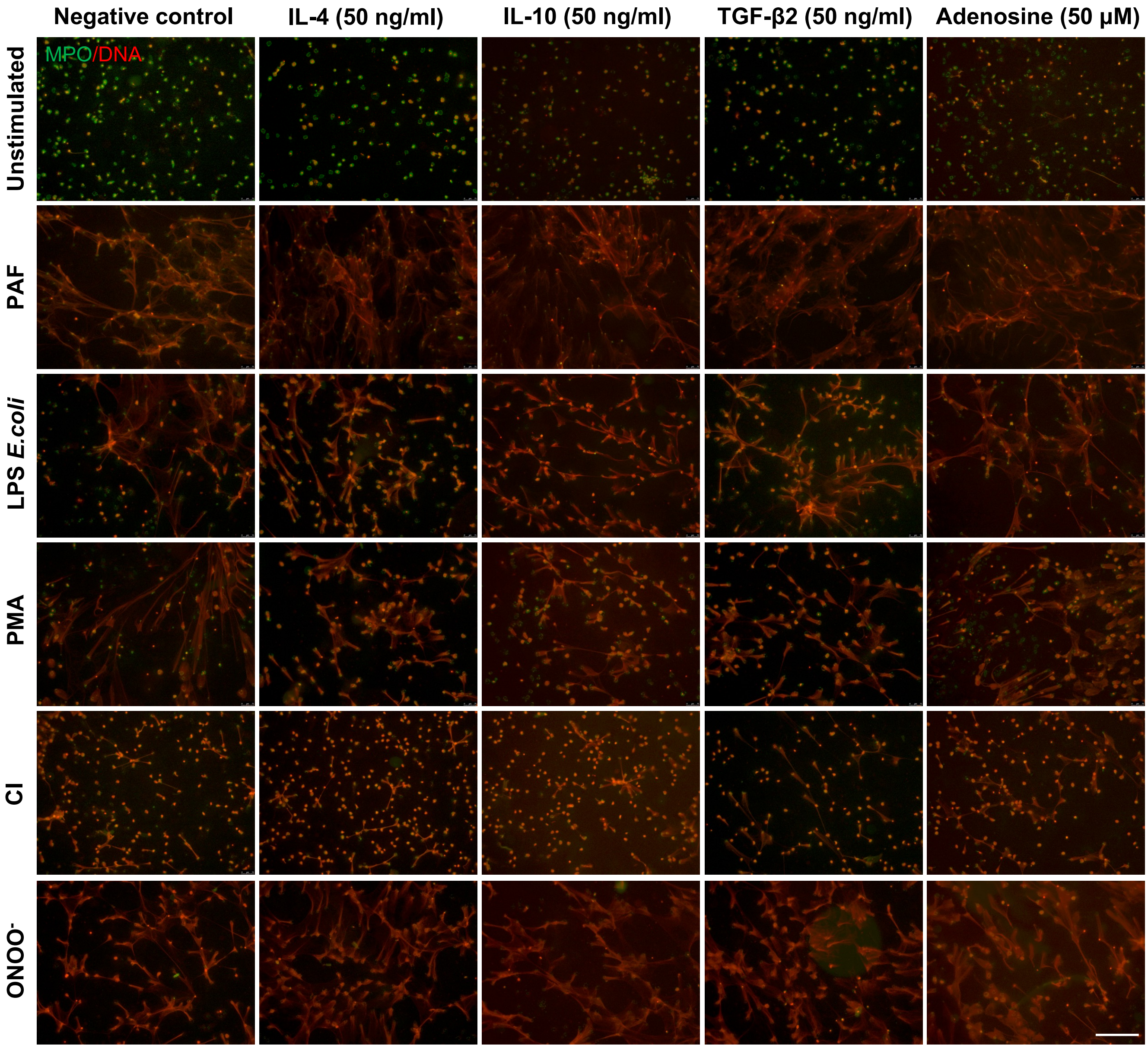

Supplementary Fig. 1 Interleukin (IL) 4 , IL-10, transforming growth factor β (TGF-β) nor adenosine do not affect NET release. Neutrophils were incubated with indicated concentrations of IL-4, IL-10, TGF-β or adenosine for 1 hour. Neutrophils cultured without addition of cytokines constituted negative control (neg ctrl). The cells were then stimulated with 2.5 µM platelet–activating factor (PAF), 100 µM peroxynitrtite ($ONOO^-$), 4 µM calcium ionophore A23187 (CI), 100 nM phorbol 12-myristate 13-acetate (PMA) or 5 µg/mL lipopolysaccharides (LPS) isolated from *E. coli*. After 3 hours, neutrophil extracellular traps (NETs) formation was assessed microscopically. Representative images of one out of 3 experiments using various blood donors are shown. Bar – 250 µm.

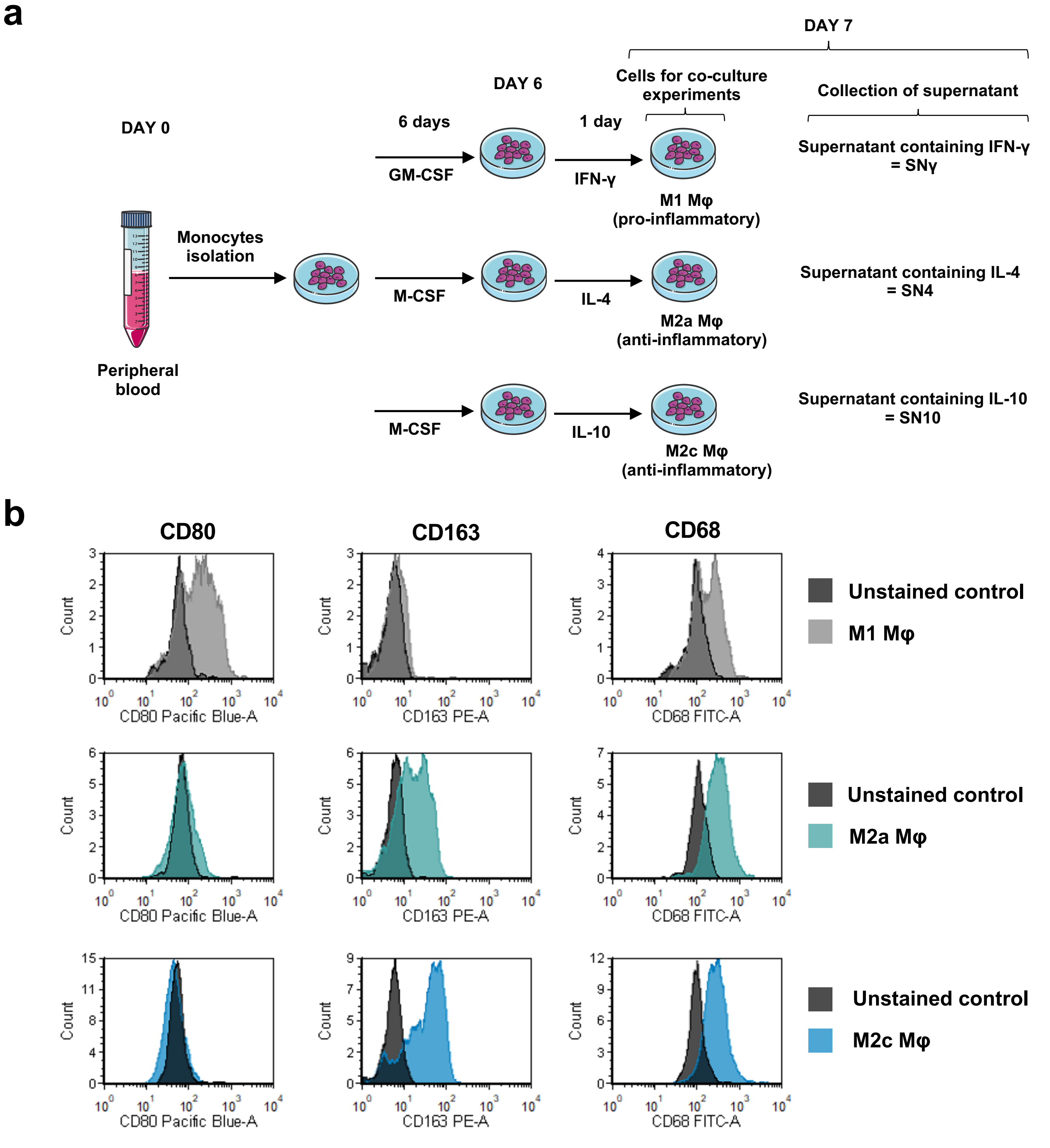

Supplementary Fig. 2 (**a**) Schematic representation of the protocol of monocyte differentiation into M1 (pro-inflammatory) and M2 (anti-inflammatory) macrophages and collection of supernatants. Briefly, monocytes were isolated from peripheral blood of healthy donors and cultured for 6 days in RPMI-1640 medium containing 10% fetal bovine serum (FBS) and antibiotic-antimycotic solution (i.e. full RPMI-1640 medium) with the addition of 50 ng/mL granulocyte-macrophage colony-stimulating factor (GM-CSF) or 50 ng/mL macrophage colony-stimulating factor (M-CSF). On the 6th day, the cells were washed and fresh full RPMI-1640 medium containing 50 ng/mL interferon gamma (IFN-y, M1 macrophages), 20 ng/mL interleukin (IL) 4 (M2a macrophages) or 20 ng/mL IL-10 (M2c macrophages) was added. Next day, the cells were harvested for co-culture experiments. Alternatively, the cells were washed on the 6th day and RH medium (FBS-free RPMI-1640 medium supplemented with 10 mM HEPES buffer) containing IFN-y, IL-4 or IL-10 was added to the cells and after 24 hours, supernatant was collected. (**b**) Expression of CD80, CD163, and CD68 was assessed by flow cytometry after 7 days of differentiation, histograms show representative data from one out of 2 experiments using monocytes from unique blood donors. *Mφ* macrophages.

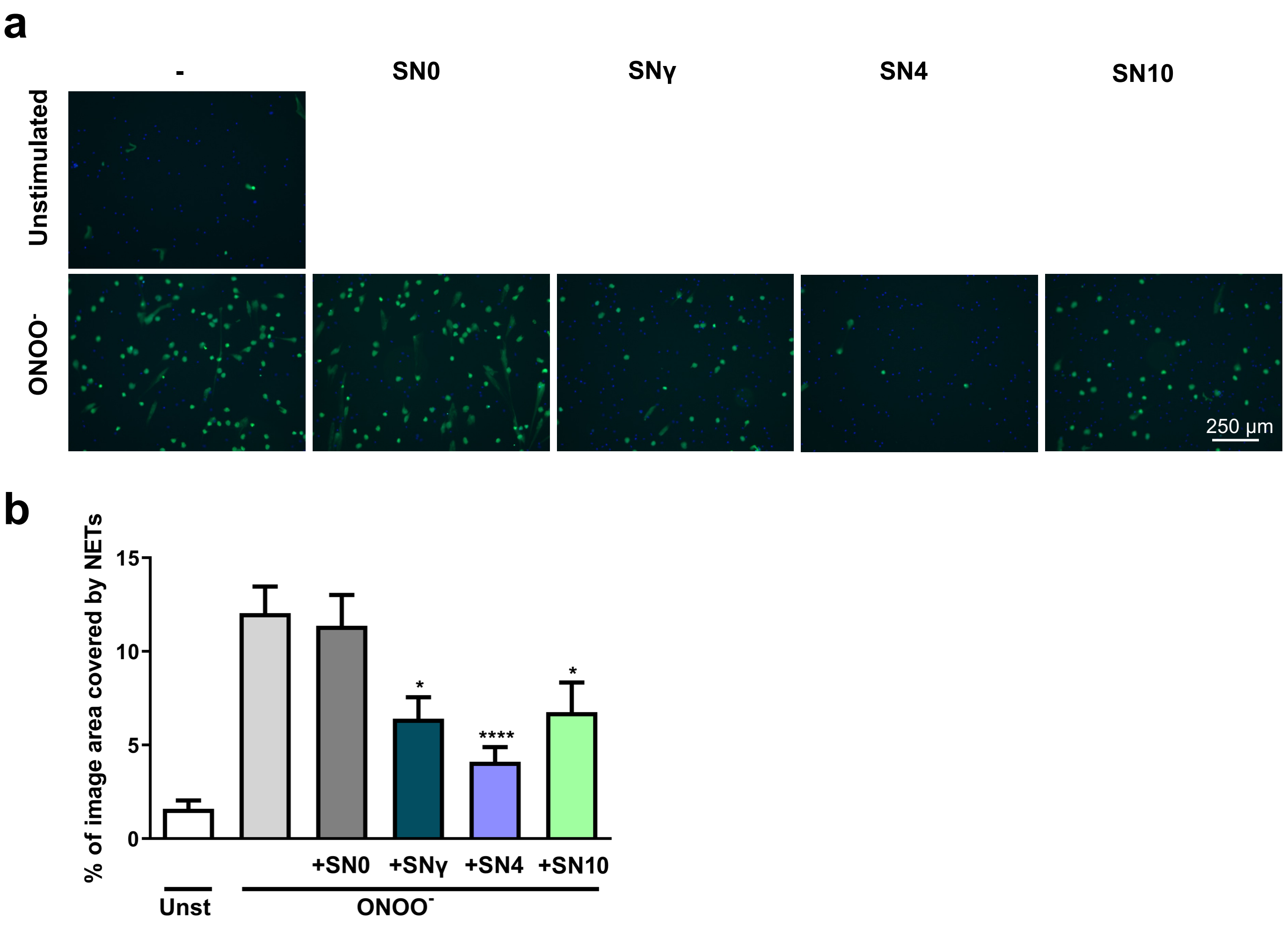

Supplementary Fig. 3 Secretome of unpolarized macrophages does not affect NET release. Neutrophils were incubated for 1 hour with supernatant collected from monocytes differentiated into M0, M1, M2a or M2c macrophages. Collected supernatants contained cytokines that were used for the polarization of macrophages, i.e. 50 ng/mL IFN-γ (M1), 20 ng/mL IL-4 (M2a) or IL-10 (M2c) and were abbreviated as SNγ, SN4 or SN10, respectively. SN0 was collected from the samples containing unpolarized macrophages, i.e. macrophages derived from monocytes cultured for 7 days in full medium without addition of any growth factors or cytokines. Alternatively, neutrophils were incubated in RH medium alone, without any supernatant. Subsequently, neutrophils were stimulated with 100 µM $ONOO^-$ and NET formation was assessed after 3 hours. To visualize NETs, DNA was stained with 1.25 µg/mL Hoechst 33342 (blue) and 100 nM SYTOX Green (green) and microscopical images were taken. Percentage coverage of image area by NETs was assessed with the use of PartSeg software with Trapalyzer Plugin. At least three images at the magnification of 10 × were taken per each condition for each biological replicate; n = 8 where n is the number of biological replicates analyzed. (**a**) Representative images are shown. (**b**) Means + SEM are shown, the data were analyzed *vs* $ONOO^-$ incubated with medium alone by Friedman test with *post-hoc* Dunn's multiple comparisons test. *P ≤ 0.05, ****P ≤ 0.0001.

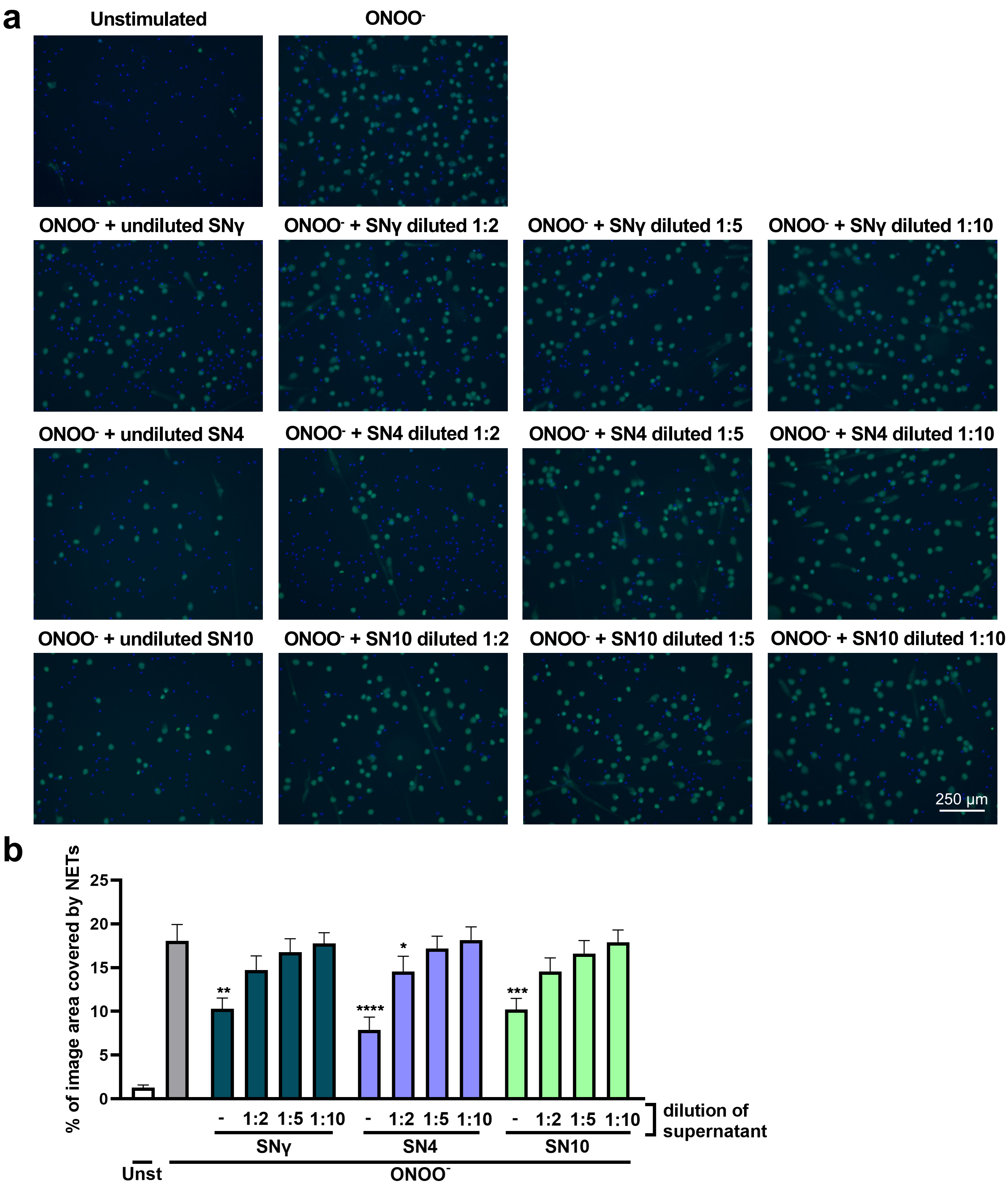

Supplementary Fig. 4 Secretome of M2a macrophages more potently diminishes NET formation than secretomes of M1 and M2c macrophages. Neutrophils were incubated for 1 hour with supernatants (undiluted or diluted in RH medium as indicated) collected from monocytes differentiated into M1, M2a or M2c macrophages. Collected supernatants contained cytokines that were used for the polarization of macrophages, i.e. 50 ng/mL IFN-γ (M1), 20 ng/mL IL-4 (M2a) or IL-10 (M2c), and were abbreviated as SNγ, SN4 or SN10, respectively. Alternatively, neutrophils were incubated in RH medium alone, without any supernatant. Subsequently, neutrophils were stimulated with 100 μM $ONOO^-$ and NET formation was assessed after 3 hours. To visualize NETs, DNA was stained with 1.25 μg/mL Hoechst 33342 (blue) and 100 nM SYTOX Green (green) and microscopical images were taken. Percentage coverage of image area by NETs was assessed with the use of PartSeg software with Trapalyzer Plugin. At least three images at the magnification of 10 × were taken per each condition for each biological replicate; n = 11. (**a**) Representative images are shown. (**b**) Means + SEM are shown, the data were analyzed *vs* $ONOO^-$ incubated with medium alone by one-way ANOVA with *post-hoc* Dunnett's multiple comparisons test. $^*P \leq 0.05$, $^{**}P \leq 0.01$, $^{***}P \leq 0.001$, $^{****}P \leq 0.0001$.

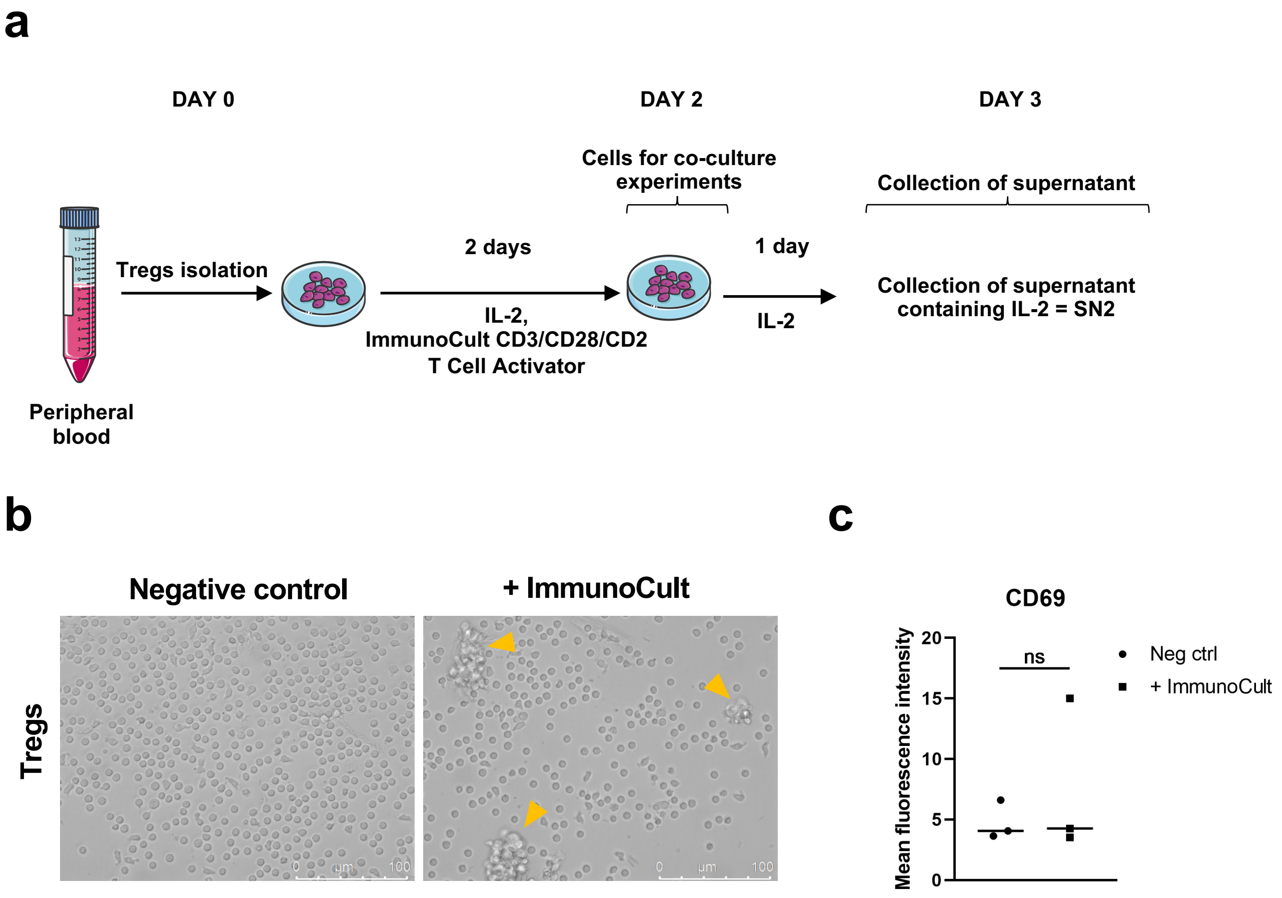

Supplementary Fig. 5 (**a**) Schematic representation of the protocol of regulatory T cells (Tregs) activation and collection of supernatant. Briefly, Tregs were isolated from peripheral blood of healthy donors and cultured for 2 days in full RPMI-1640 medium with the addition of 100 IU/mL IL-2 and ImmunoCult™ Human CD3/CD28/CD2 T Cell Activator. On the 2nd day, the cells were used for co-culture experiments. Alternatively, on the 2nd day, the cells were washed and medium was replaced with RH medium containing IL-2. After 24-hour incubation supernatants were collected. (**b**) Activation and expansion of Tregs after 2-day activation was analyzed microscopically as the formation of aggregates of activated cells (yellow arrowheads) in the presence of ImmunoCult. Negative control (neg ctrl) – Tregs cultured solely in the presence of IL-2, without addition of ImmunoCult activator. (**c**) Flow cytometry analysis of the expression of CD69 after 2-day activation. Medians with individual values are shown. Data were analyzed with paired t-test, $^{ns}P > 0.05$.

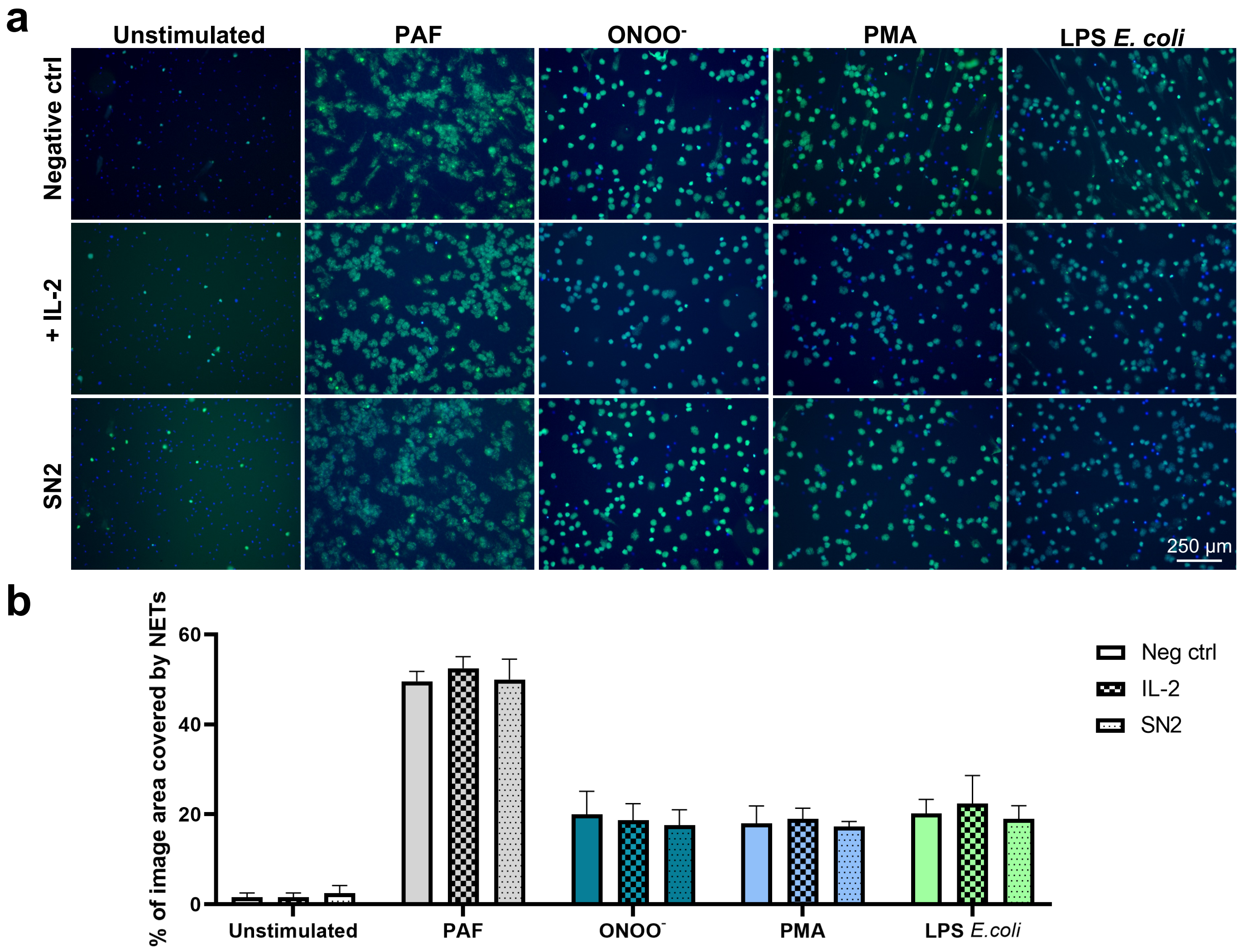

Supplementary Fig. 6 Secretome of regulatory T cells (Tregs) does not affect NET release. Tregs were activated with the use of CD3/CD2/CD28 T cell activator and subsequently cultured in the presence of IL-2. Supernatant containing IL-2, therefore abbreviated as SN2, was collected and added to neutrophils for 1-hour incubation. As controls, neutrophils were incubated in medium (negative control, neg ctrl) or in medium with IL-2. Next, the neutrophils were stimulated with 2.5 µM PAF, 100 µM $ONOO^-$, 100 nM PMA, or 5 µg/mL LPS isolated from *E. coli*, and NET formation was assessed after 3 hours. (**a**) To visualize NETs, DNA was stained with 1.25 µg/mL Hoechst 33342 (blue) and 100 nM SYTOX Green (green) and microscopical images were taken. At least two images at the magnification of 10 × were taken per each condition for each biological replicate. (**b**) Percentage coverage of image area by NETs was assessed with the use of Parteg software with Trapalyzer Plugin. N = 4. (**a**) Representative images are shown. (**b**) Means + SEM are shown, for each stimulus the data were analyzed *versus* negative control by one-way ANOVA (unstimulated, PAF, $ONOO^-$, PMA) or Friedman test (LPS *E. coli*).

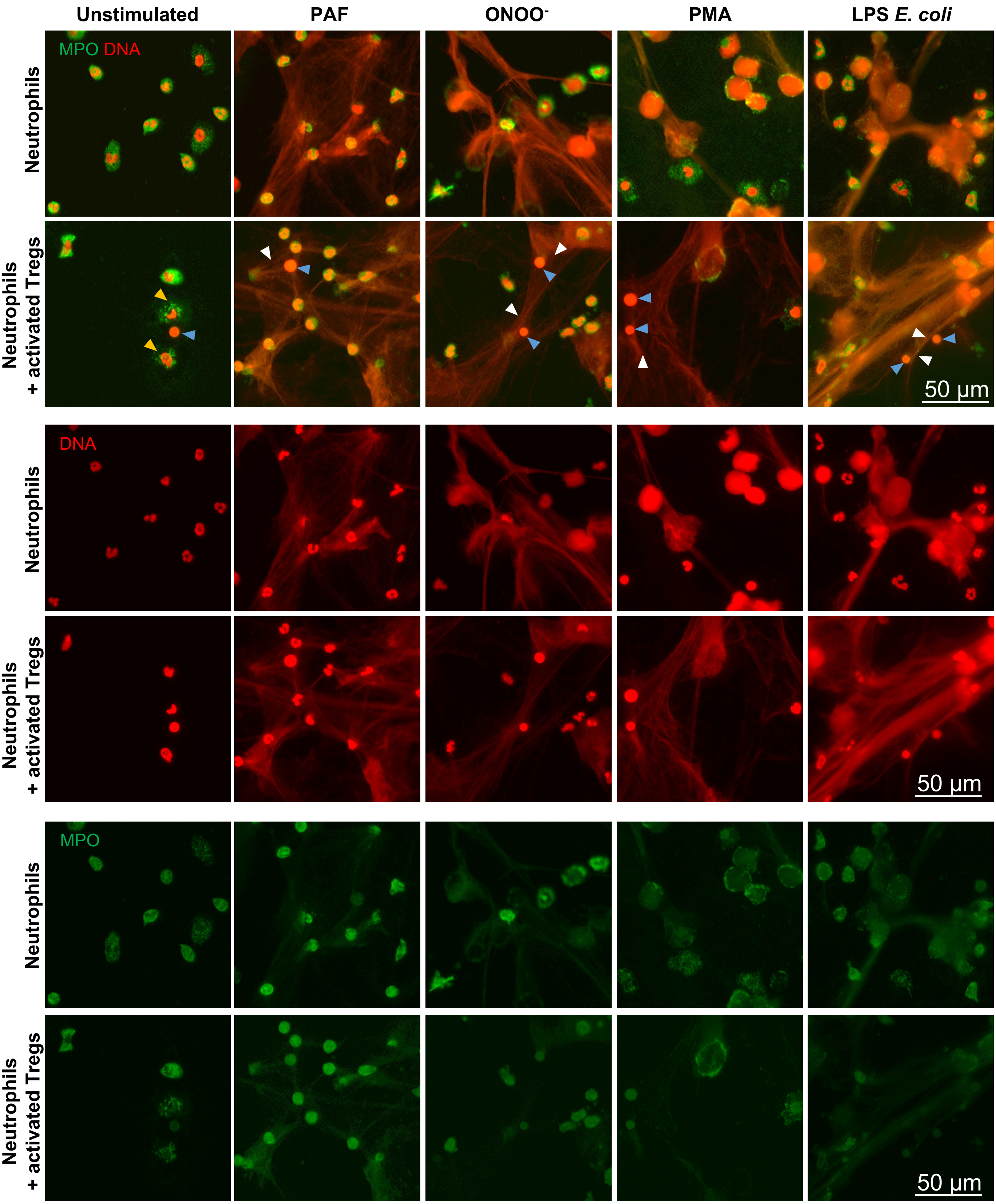

Supplementary Fig. 7 Co-culture of Tregs with neutrophils does not affect NET release. Tregs were pre-activated with CD3/CD28/CD2 T Cell Activator and then co-cultured with neutrophils at the ratio 10:1 (neutrophils:Tregs) for 2 hours. Neutrophils were stimulated with 2.5 µM PAF, 100 µM $ONOO^-$, 100 nM PMA or 5 µg/mL LPS isolated from *E. coli* to release NETs. Three hours post stimulation the samples were fixed with paraformaldehyde and neutrophils were immunostained with anti-MPO antibody (green color) and DNA was counterstained with SYTOX Orange (red). Accordingly, granulocytes are stained with green and orange dyes, whereas Tregs'cytoplasm does not stain with green. Merge images (top) and single channels for DNA (middle) and MPO (down) are shown. Blue arrowheads point to Tregs. Yellow arrowheads point to resting granulocytes adjacent to Tregs. White arrowheads point to NET-derived DNA adjacent to Tregs. Images are representative for four biological replicates.

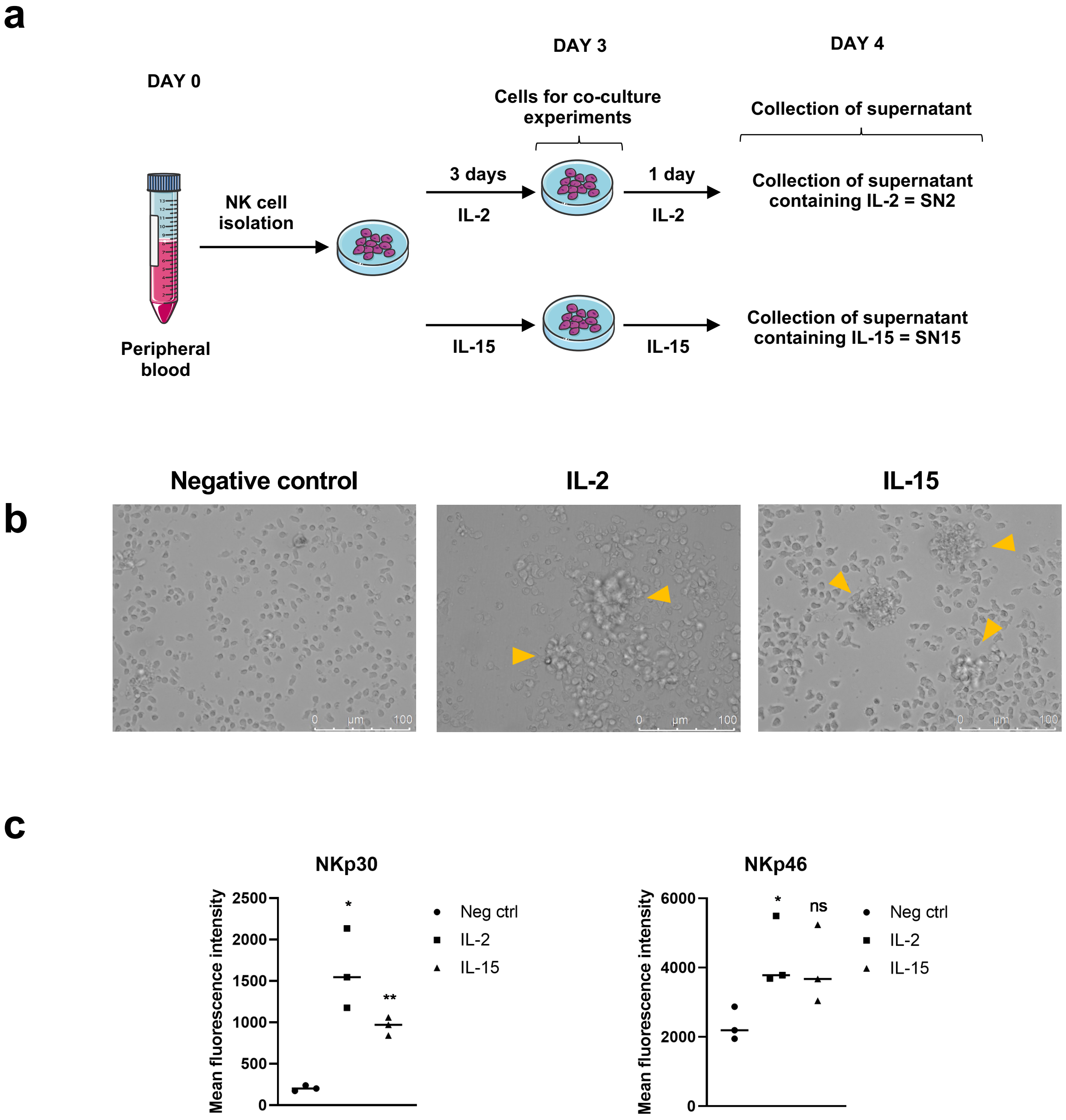

Supplementary Fig. 8 (**a**) Schematic representation of the protocol of NK cell activation. Briefly, NK cells were isolated from peripheral blood of healthy donors and cultured for 3 days in full RPMI-1640 medium with the addition of 100 IU/mL IL-2 or 10 IU/mL IL-15. On the 3rd day, the cells were used for co-culture experiments. Alternatively, on the 3rd day the cells were washed and medium was replaced with RH medium and fresh cytokines were added. After 24-hour incubation supernatants were collected. (**b**) Activation and expansion of NK cells was observed microscopically as the formation of aggregates of activated cells (yellow arrowheads). Negative control (neg ctrl) – NK cells cultured without addition of IL-2 or IL-15 cytokines. (**c**) Activation of NK cells was analyzed by flow cytometry as the increase in the expression of NKp30 and NKp46 activating receptors. Medians with individual values are shown. Data were analyzed with one-way ANOVA with *post-hoc* Dunnett's test. $^{Ns}P > 0.05$, $^{*}P \leq 0.05$, $^{**}P \leq 0.01$ *vs* neg ctrl.

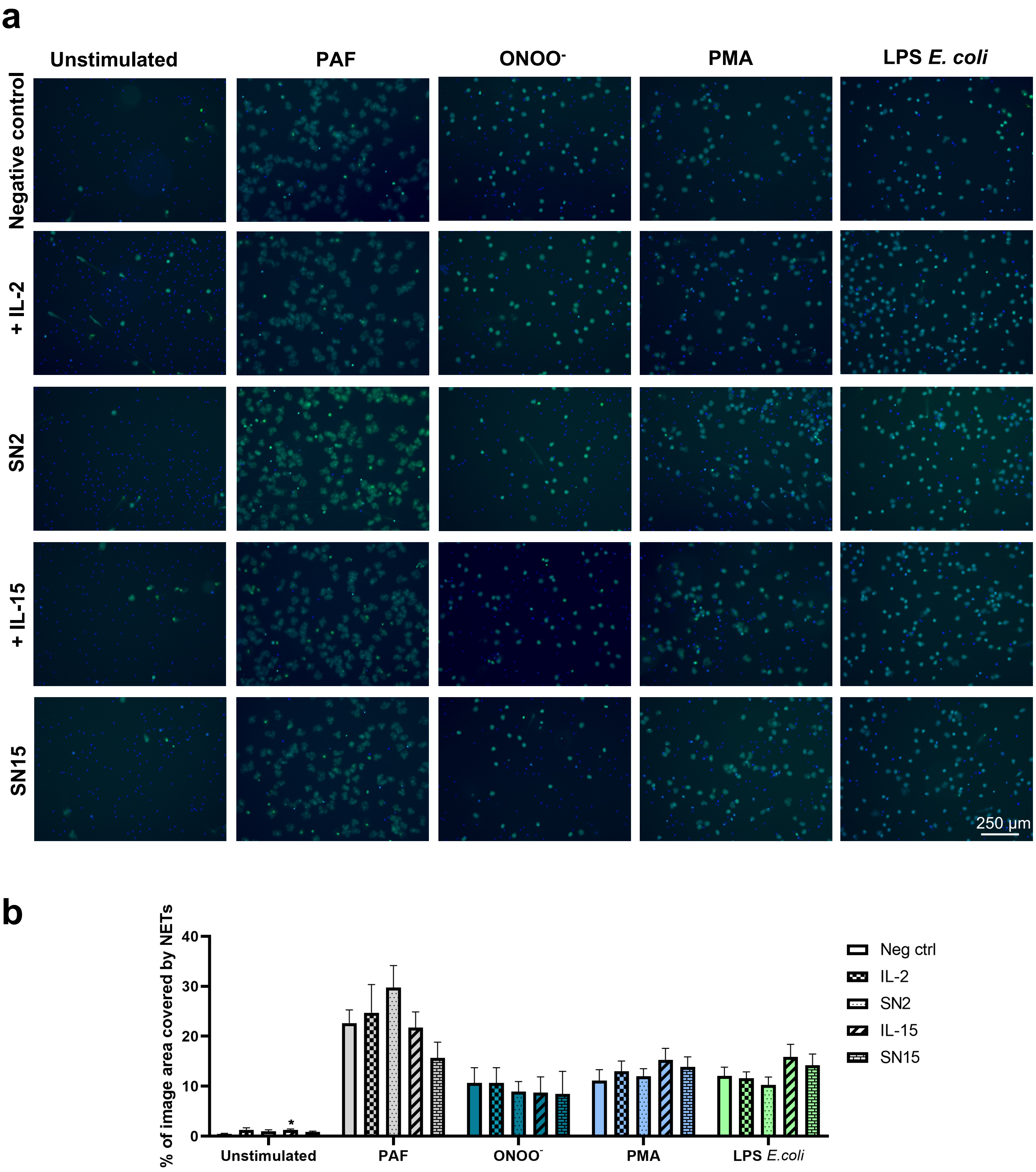

Supplementary Fig. 9 Secretome of NK cells does not affect NET release. Neutrophils were incubated for 1 hour with supernatant collected from activated NK cells. These supernatants contained cytokines that were used to activate NK cells, IL-2 or IL-15, and were abbreviated as SN2 and SN15, respectively. As controls, neutrophils were incubated in medium (negative control, neg ctrl) or in medium with IL-2 or IL-15. Subsequently, neutrophils were stimulated with 2.5 µM platelet activating factor (PAF), 100 µM peroxynitrtite ($ONOO^-$), 100 nM phorbol 12-myristate 13-acetate (PMA), or 5 µg/mL LPS isolated from *E. coli.* NET formation was assessed after 3 hours. (**a**) To visualize NETs, DNA was stained with 1.25 µg/mL Hoechst 33342 (blue) and 100 nM SYTOX Green (green) and microscopical images were taken. Routinely, three images at the magnification of 10 × were taken per each condition for each biological replicate. (**b**) Percentage coverage of image area by NETs was assessed with the use of PartSeg software with Trapalyzer Plugin. N = 6. (**a**) Representative images are shown. (**b**) Means + SEM are shown, for each stimulus data were analyzed *versus* negative control by Friedman test with *post-hoc* Dunn's test when appropriate, * $P \leq 0.05$.

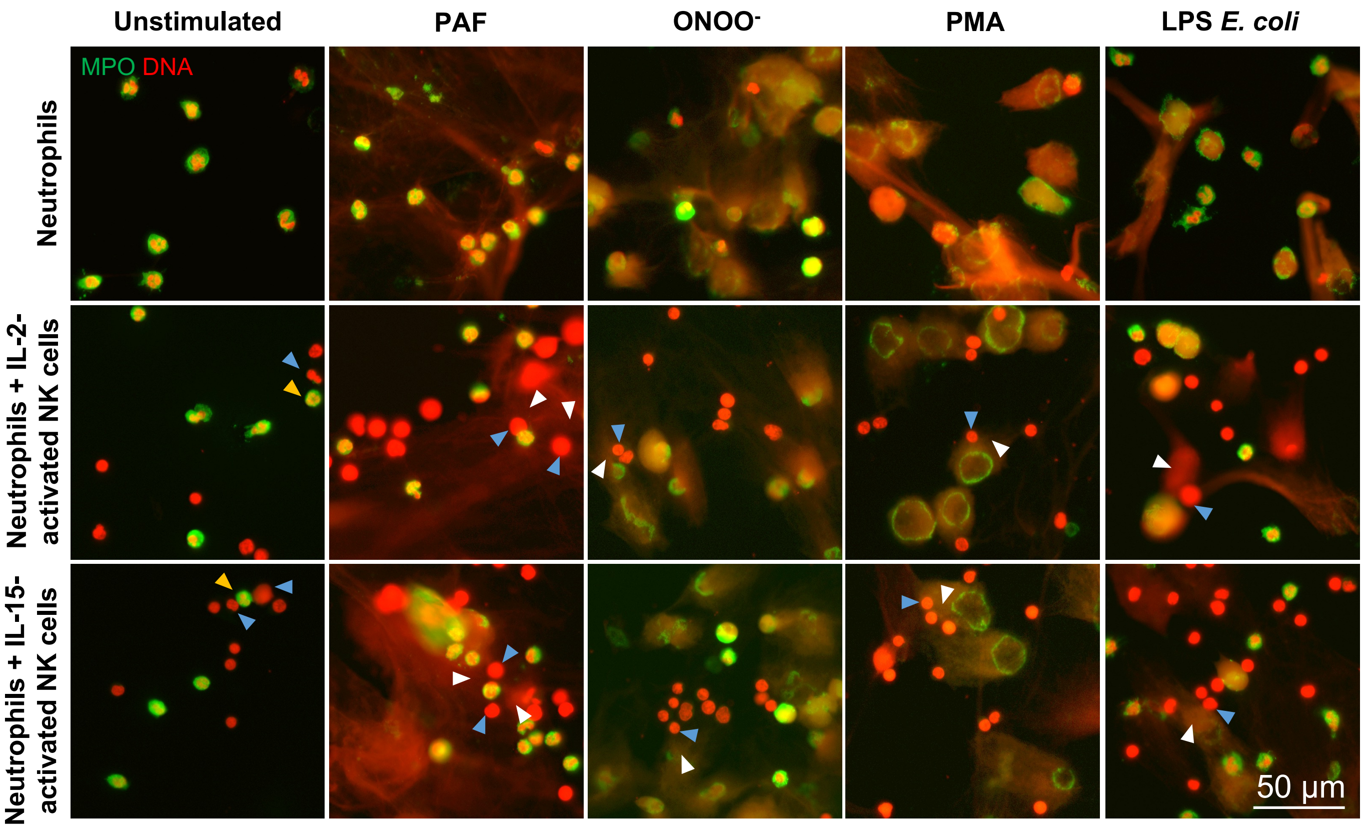

Supplementary Fig. 10 Co-culture of NK cells with neutrophils does not affect NET release. NK cells were pre-activated with IL-2 or IL-15 for 3 days. Subsequently, neutrophils were isolated and co-cultured with NK cells at the ratio 1:1 for 1 hour and then neutrophils were stimulated with 2.5 µM PAF, 100 µM $ONOO^-$,100 nM PMA or 5 µg/mL LPS isolated from *E. coli* to release NETs. Three hours after the stimulation, the samples were fixed with paraformaldehyde and neutrophils were immunostained with anti-MPO antibody (green color) and DNA was counterstained with SYTOX Orange (red). Accordingly, granulocytes are stained with green and orange dyes, whereas NK cells' cytoplasm does not stain with green. Blue arrowheads point to NK cells. Yellow arrowheads point to multilobulated granulocytes adjacent to NK cells. White arrowheads point to NET-derived DNA adjacent to NK cells. Images are representative for two (IL-15-activated NK cells) or three (IL-2-activated NK cells) biological replicates.

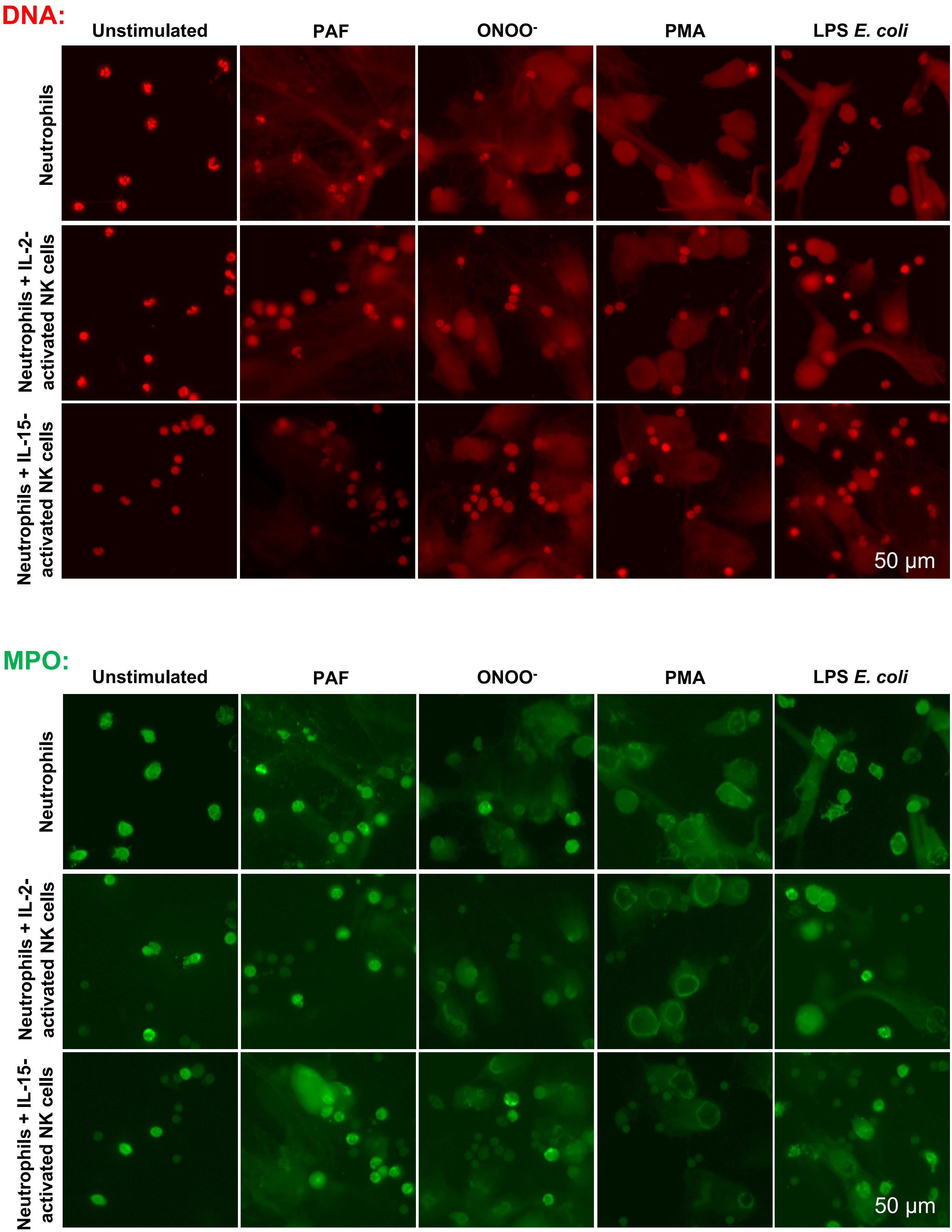

Supplementary Fig. 11 Co-culture of NK cells with neutrophils does not affect NET release. Single-channel images, for which merged images are presented in Supplementary Fig. 10, are shown in this figure.
